# Accelerated differentiation of neo-W nuclear-encoded mitochondrial genes between two climate-associated bird lineages signals potential co-evolution with mitogenomes

**DOI:** 10.1038/s41437-024-00718-w

**Published:** 2024-08-22

**Authors:** Gabriel Weijie Low, Alexandra Pavlova, Han Ming Gan, Meng-Ching Ko, Keren R. Sadanandan, Yin Peng Lee, J. Nevil Amos, Lana Austin, Stephanie Falk, Damian K. Dowling, Paul Sunnucks

**Affiliations:** 1 School of Biological Sciences, Monash University, Melbourne, VIC 3800 Australia; 2 Evolution of Sensory and Physiological Systems, Max Planck Institute for Biological Intelligence, 82319 Seewiesen, Germany; 3 National Parks Board, 1 Cluny Road, Singapore Botanical Gardens, Singapore, 259569 Singapore; 4 Deakin Genomics Centre, Deakin University, Geelong, VIC 3220 Australia; 5 Patriot Biotech Sdn Bhd, 47500 Subang Jaya, Selangor Malaysia; 6 School of Life and Environmental Sciences, Deakin University, Geelong, VIC 3220 Australia; 7 Arthur Rylah Institute for Environmental Research, Heidelberg, VIC 3084 Australia; 8 Max Planck Institute of Immunobiology and Epigenetics, 79108 Freiburg, Germany

**Keywords:** Evolutionary genetics, Evolutionary biology, Genetic variation, Speciation, Comparative genomics

## Abstract

There is considerable evidence for mitochondrial-nuclear co-adaptation as a key evolutionary driver. Hypotheses regarding the roles of sex-linkage have emphasized Z-linked nuclear genes with mitochondrial function (N-mt genes), whereas it remains contentious whether the perfect co-inheritance of W genes with mitogenomes could hinder or facilitate co-adaptation. Young (neo-) sex chromosomes that possess relatively many N-mt genes compared to older chromosomes provide unprecedented hypothesis-testing opportunities. Eastern Yellow Robin (EYR) lineages in coastal and inland habitats with different climates are diverged in mitogenomes, and in a ~ 15.4 Mb nuclear region enriched with N-mt genes, in contrast with otherwise-similar nuclear genomes. This nuclear region maps to passerine chromosome 1A, previously found to be neo-sex in the inland EYR genome. To compare sex-linked Chr1A-derived genes between lineages, we assembled and annotated the coastal EYR genome. We found that: (i) the coastal lineage shares a similar neo-sex system with the inland lineage, (ii) neo-W and neo-Z N-mt genes are not more diverged between lineages than are comparable non-N-mt genes, and showed little evidence for broad positive selection, (iii) however, W-linked N-mt genes are more diverged between lineages than are their Z-linked gametologs. The latter effect was ~7 times stronger for N-mt than non-N-mt genes, suggesting that W-linked N-mt genes might have diverged between lineages under environmental selection through co-evolution with mitogenomes. Finally, we identify a candidate gene driver for divergent selection, NDUFA12. Our data represent a rare example suggesting a possible role for W-associated mitochondrial-nuclear interactions in climate-associated adaptation and lineage differentiation.

## Introduction

Understanding the mechanisms underlying climate adaptation can help predict changes in individual fitness, population dynamics and species distributions, and thus improve biodiversity management under climate change (Franks and Hoffmann 2012; Gienapp et al. 2008; Sandvig et al. 2017). There is growing recognition of the importance in climate adaptation of mitochondrial phenotypes, such as the efficiency with which individuals harness energy from nutrients, and the amount of metabolic heat and reactive oxygen species generated during cellular respiration (Koch et al. 2021; Lasne et al. 2018; Sunnucks et al. 2017). Many mitochondrial phenotypes are outcomes of complex interactions between mitochondrial protein complexes jointly encoded by the mitochondrial (mt) and nuclear genomes, and are under strong selection associated with environmental factors including temperature, moisture and food availability (Dowling et al. 2008; Morales et al. 2015). Emerging studies suggest that climate-associated selection can drive mitonuclear co-evolution, involving the co-tuning of mitochondrial genes, nuclear-encoded genes with mitochondrial function (N-mt genes), and the specific combinations of mitochondrial and N-mt genes (Bar-Yaacov et al. 2012; Burton et al. 2013; Dowling and Adrian 2019; Hill et al. 2019; Lamb et al. 2018; Sloan et al. 2017; Sunnucks et al. 2017). Hybridization between isolated populations with different locally-adapted mitonuclear genotypes may result in fitness loss in hybrids bearing incompatible or suboptimal combinations of mitochondrial and N-mt alleles (i.e. mitonuclear incompatibilities), which could contribute to barriers to gene flow and promote lineage differentiation (Ågren et al. 2019; Biot-Pelletier et al. 2023; Burton and Barreto 2012; Burton et al. 2013). However, demonstrations that climate-associated selection drives divergence of mitonuclear genotypes in the presence of gene flow are still rare (Wang et al. 2021).

Genomic architecture that links the inheritance of multiple N-mt genes may facilitate co-evolution of suites of N-mt genes with the mitochondrial genome (Sunnucks et al. 2017). Sex chromosomes may be well-positioned to promote mitochondrial-nuclear (mitonuclear) co-evolution because they experience suppressed recombination and have lower effective population sizes (*N*_*e*_), and are expected to evolve faster than autosomes. Empirical observations of faster evolution on Z-linked chromosomes have been hypothesized to allow N-mt genes to keep pace with rapid mitogenome evolution (which is >7 times faster than autosomal evolution in birds), thereby facilitating mitonuclear co-evolution (Allio et al. 2017; Hill 2014). There has been strong emphasis in taxa with ZW sex-determination, including birds and butterflies, on the role of Z-linked N-mt genes as potential drivers of key aspects of evolutionary ecology, including sexual selection, climate-associated adaptation and speciation (Hill and Johnson 2013; Hill 2019). However, some key concepts associated with hypotheses concerning sex-linkage of N-mt genes may be in conflict, notably that while co-inheritance of N-mt genes and mitogenomes may reinforce the inheritance of co-adapted genotypes, this lockstep mode of evolution could also inherently reduce the efficiency of individual selection on each of these separate genome elements (Hill 2014). In ZW taxa, the mitochondrial genome (mitogenome) and W chromosome are completely co-inherited. In contrast to a main emphasis on Z-linkage, it has also been hypothesized that W-mitogenome co-inheritance could provide a pathway for selection to preserve optimal combinations of alleles spanning mitochondrial and nuclear genomes (Berlin et al. 2007; Irwin 2018). However, testing these hypotheses has been challenging given a scarcity of study models with appreciable numbers of Z- and W-linked N-mt genes.

Sex chromosomes are more susceptible to genetic drift and accumulation of deleterious mutations under weakened purifying selection than are autosomes (Charlesworth et al. 2000). These effects are particularly strong in haploid chromosomes with sex-limited inheritance (the female-limited W and male-limited Y chromosomes) and correspondingly even lower *N*_*e*_, leading to gene degradation and loss on old sex chromosomes (Berlin and Ellegren 2006; Irwin 2018; Mank and Ellegren 2007). However, some species have sex chromosomes that are relatively young (*neo*-sex chromosomes), as a result of recent fusions of former autosomes to sex chromosomes (Bachtrog 2006; Bracewell et al. 2017; Gan et al. 2019; Wright et al. 2016). Sex-linked genes on neo-sex chromosomes are therefore more likely to remain functional, and genes on haploid sex chromosomes in particular would tend to be under very strong sex-specific selection; for example, in ZW sex determination systems, neo-W genes could be strongly influenced by female fitness (Connallon and Clark 2010; Mank 2012). ZW neo-sex chromosome architecture has been reported in butterflies (Smith et al. 2016) and non-avian reptiles (Augstenová et al. 2018; Rovatsos et al. 2019). Increasingly, extensive ZW neo-sex chromosome systems are being detected in birds, for example involving chromosomes 3, 4A, and 5 among others in Sylvioid warblers and allies (superfamily Sylvioidea), chromosome 5 in honeyeaters (family Meliphagidae), chromosomes 11 and 25 in parrots (order Psittaciformes), and chromosome 1A in the Eastern Yellow Robin (family Petroicidae) (Burley et al. 2022; Gan et al. 2019; Huang et al. 2022; Pala et al. 2012a; Pala et al. 2012b; Sigeman et al. 2020).

Given their near-identical genealogies, we might expect any beneficial alleles that arise in the mitochondrion or neo-W chromosome as a result of local environment-driven selection to rapidly sweep both to fixation. In ZW sex-determined species, this could therefore represent an avenue for females to adapt to their respective environments in a way that is unavailable to males (which do not possess W chromosomes). Females that adapt better to local environments in this manner would be expected to contribute less to overall gene flow between lineages and therefore promote divergence between lineages. In addition, it is widely accepted that dosage-sensitive and highly-conserved genes (such as those with mitochondrial functions) are most likely to retain functionality the longest on degrading W chromosomes, even as mildly deleterious alleles accumulate on nearby genes because of the low effective population size of this chromosome (Al-Ajli et al. 2023; Sigeman et al. 2021; Smeds et al. 2015; Wright et al. 2016; Xu and Zhou 2020). Otherwise advantageous mitochondrial DNA or neo-W-driven sweeps in independently evolving lineages may therefore allow co-sweeps of mildly deleterious neo-W alleles. This could lead to the formation of Bateson-Dobzhansky-Muller incompatibilities between mtDNA and neo-W chromosomes that manifest in the genomic backgrounds of other lineages upon subsequent secondary contact and hybridization, thereby contributing to gene flow barriers between them (Irwin 2018).

Neo-sex chromosomes in the Eastern Yellow Robin (*Eopsaltria australis*; EYR), a small passerine, involve a notable over-representation of sex-linked N-mt genes that have been flagged as potential targets for climate-driven evolution of mitonuclear interactions (Morales et al. 2018). Across its range in eastern Australia, this species shows genome-wide differentiation into northern and southern populations, but also geographically orthogonal inland-coastal differentiation in mitogenomes ( ~ 6% mitolineage nucleotide sequence divergence) and in a ~ 15.4 Mb region of nuclear genome homologous to chromosome 1A of zebra finch and other passerines, enriched for N-mt genes (Morales et al. 2017; Morales et al. 2018; Pavlova et al. 2013). Population-level analysis of mitogenomes revealed three genes, encoding parts of oxidative phosphorylation (OXPHOS) complexes I and IV (ND4 and ND4L and cyt-b), that showed results consistent with divergence under positive selection between inland and coastal lineages (Morales et al. 2015). Additionally, five of the 32 N-mt genes located in the 15.4-Mb region were identified as potential candidates for interacting with these three mtDNA genes to affect structure and function of Complex I (FMC1, NDUFA6, NDUFA12, and NDUFB2) and mitochondrial regulation (YARS2) (Morales et al. 2018). The inland EYR lineage, occurring in warmer, drier, more thermally variable habitat, and the coastal EYR lineage in cooler, wetter and more thermally consistent habitat, are still connected at regional scales by male-mediated nuclear gene flow, notwithstanding the occurrence of inter-lineage hybrids of both sexes in the intermediate environment of the contact zone (Morales et al. 2017; Pavlova et al. 2013). It was hypothesized that climate-associated selection drove mitonuclear divergence between inland and coastal lineages (Morales et al. 2018; Pavlova et al. 2013). In the inland EYR lineage, the differentiated 15.4-Mb region formerly belonging to autosome 1A is inherited in a neo-sex fashion due to fusion with ancestral Z and/or ancestral W chromosomes (Gan et al. 2019). Coalescent analyses of nuclear and mitochondrial sequences indicated two introgressions between inland and coastal eastern yellow robin lineages, of mitogenomes and the associated 15.4-Mb region now known to be sex-linked (Morales et al. 2017; Gan et al. 2019). It seems parsimonious from this observation that the two lineages might have homologous neo-sex chromosome systems, but this remains untested even though the presence or absence of a neo-sex chromosome 1A in the coastal lineage greatly affects our interpretation of how mitonuclear divergence arose in these two lineages.

In this study, we sequenced, assembled and annotated the genome of the coastal EYR lineage to investigate its genomic architecture. We then compared inland and coastal EYR lineages to test three hypotheses. H1: The coastal lineage has a neo-sex arrangement homologous to that of the inland lineage. H2: Co-evolution of N-mt genes on neo-sex chromosomes with the mitogenome within each EYR lineage leads to their accelerated evolution due to the necessity of maintaining functional mitonuclear phenotypes as each lineage’s mitogenome rapidly evolves. This leads to divergent evolution driven by local adaptation of mitonuclear phenotype, hybrid incompatibility, or both, and could result in proportionally more neo-sex N-mt genes than non-N-mt genes being under divergent selection between lineages. H3: Co-inheritance of mitogenomes and W-linked N-mt genes promotes their rapid co-evolution in one or both lineages. Under this hypothesis, we would predict stronger differentiation between lineages in neo-W gametologs of N-mt genes than in their respective neo-Z gametologs, in excess of divergence resulting from genetic drift. This effect should therefore be stronger for N-mt than non-N-mt genes, and be reflected in tests for molecular selection.

## Materials and methods

**Fig. 1:**
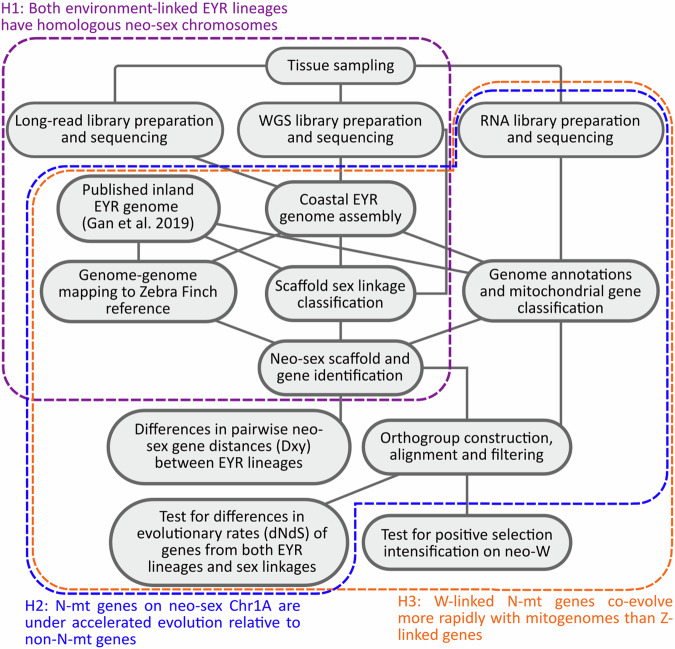
The workflow in this study is summarized in this schematic. Each different coloured background outlines the steps taken to address a given hypothesis. (H1, purple) Identification and confirmation of neo-sex Chr1A behaviour in the coastal Eastern Yellow Robin (EYR) would mirror that seen in the published inland EYR genome and indicate that neo-sex Chr1A arose before the two lineages split. (H2, blue) Identification and classification of Chr1A-derived neo-sex genes into bins with or without mitochondrial functions (N-mt versus non-N-mt genes), followed by testing for differences in protein evolution rates (dNdS) and differentiation levels between lineages (Dxy) would show whether neo-sex N-mt genes are undergoing accelerated evolution relative to non-N-mt genes. (H3, orange) A similar comparison of neo-W versus neo-Z Chr1A-derived genes allows us to draw conclusions about the relative rates of evolution on each neo-sex chromosome, supplemented by an additional search for neo-W candidates under intensified positive selection possibly resulting from accelerated co-evolution with mitochondrial DNA.

This research was conducted under wildlife permits 10009243 under the Wildlife Act 1975 and National Parks Act 1975, and NW11047F under the Forests Act 1958. All live bird handling was regulated under Australian Bird and Bat Banding Scheme Authority No. 2870. Animal ethics authorization was given through Monash University under BSCI 2015 20. The methods described in this section are visually summarized in Fig. 1.

### Tissue sampling and DNA/RNA sequencing

To obtain a female genome assembly for the coastal EYR lineage, we sampled blood from a female EYR, VIC030 (ABBBS band number 027-09430), captured in coastal habitats near Vaughan, Victoria, Australia in 2017 (latitude −37.17, longitude 144.22), as described in Morales et al. (2018). ND2 sequencing placed this individual within the coastal mtDNA lineage (mito-B; NCBI Accession MH377680) (Morales et al. 2018). Whole DNA was extracted with a Qiagen DNAeasy Blood and Tissue Kit (Qiagen, Hilden, Germany). A standard paired-end Illumina library was constructed from 100 ng of QSonica-fragmented DNA ( ~ 300 bp) using the NEB Ultra Illumina Library Preparation kit (New England Biolabs, Ipswich, MA) and sequenced on an Illumina NovaSEQ 6000 (2 ×151 bp paired-end configuration; Illumina, San Diego, CA) at Deakin Genomics Centre, Australia. We obtained short-read depth coverage of ~170x. Additionally, we also generated long-read data for the VIC030 genome assembly using Oxford Nanopore and PacBio sequencing techniques (Supplementary Methods), with sequencing yields of around ~2x and ~10x coverage, respectively. For comparison with the coastal lineage, we used the published female EYR genome from the inland lineage (Voucher EYR054, NCBI Accession GCA_003426825.1) (Gan et al. 2019).

To characterize the sex-linkage of genome scaffolds in both of these genomes, we obtained whole genome resequencing (WGS) data for nine inland and nine coastal EYRs of each sex, captured in Victoria, Australia in 2017 (*n* = 36, see Supplementary Table 1) (Morales et al. 2018) using the same extraction, library preparation, and sequencing protocols as for VIC030. We obtained short-read depth coverage of ~10x for these 36 samples. We did not obtain long-read data for these samples.

To provide evidence for genome annotation, we used RNA extracted from samples of brain, muscle, liver, and eye tissues from two EYR nestlings from the coastal lineage (M19.32.1 female and M19.32.2 male; Supplementary Table 1) in 2019. Total RNA was extracted from each sampled tissue of female M19.32.1 and male M19.32.2 using the Quick-RNA MiniPrep Kit (Zymo Research, Irvine, CA) according to the manufacturer’s protocols. We pooled equimolar amounts of RNA from each tissue type for each individual, and prepared each library from 500 ng of RNA using the NEBNext® Ultra™ II Directional RNA Library Prep Kit for Illumina® (New England Biolabs, Ipswich, MA) according to the manufacturer’s protocol, except including a 5-fold dilution of the NEBNext adaptor for Illumina (provided at 15 μM) in dilution buffer (10 mM Tris-HCI, 10 mM NaCl) (Astral Scientific, Australia). After several quality assessment and read-distribution control stages (Supplementary Methods), the final pooled library was sequenced on an Illumina NovaSeq 6000 Sequencer (2× 150 bp paired end reads) (Illumina, San Diego, CA) at Deakin Genomics Centre, Australia.

### Hybrid de novo genome assembly

Raw Illumina paired-end reads were adapter- and poly-G-trimmed using fastp v0.20.0 (Chen et al. 2018). Nanopore reads were trimmed using Porechop v0.2.3 (Wick 2018). Sequencing quality metrics of both Nanopore and PacBio long-read sequencing data were generated with the NanoPack suite (De Coster et al. 2018). We used MaSuRCA v3.2.8 (Zimin et al. 2013; Zimin et al. 2017) to perform a hybrid de Bruijn and Overlap-Layout-Consensus (OLC) de novo genome assembly with all data types available for VIC030.

### Genome annotation of coastal VIC030 and inland EYR054 genomes

We prepared the newly-assembled coastal VIC030 genome as well as the already available inland EYR genome (Voucher EYR054, GenBank accession GCA_003426825.1) for structural annotation by modelling repetitive regions with RepeatModeler2 (Flynn et al. 2020) and soft-masking them with RepeatMasker v4.1.0 (Smit et al. 2015). BUSCO v3.0 (Waterhouse et al. 2017) was used to assess the completeness of the soft-masked VIC030 assembly based on the aves_odb9 protein database (Zdobnov et al. 2017), while simultaneously optimizing training parameters for genome annotation with AUGUSTUS v3.3.3 (Stanke et al. 2006; Stanke et al. 2008). We assessed quality of the VIC030 and EYR054 assemblies with QUAST (Gurevich et al. 2013).

We processed male and female coastal EYR RNAseq data for use as gene annotation evidence by adopting best-practice steps outlined by the Harvard FAS Informatics Group (https://informatics.fas.harvard.edu/best-practices-for-de-novo-transcriptome-assembly-with-trinity.html) (Supplementary Methods). We retained 240 million (98.9% retention) and 257 million (98.8% retention) read pairs for the male and female EYR RNA samples respectively, and rechecked read-quality statistics with FastQC. We then performed a two-pass alignment of male and female EYR RNA reads against the EYR054 and VIC030 genome assemblies with STAR v2.7.3a (Dobin et al. 2013) using default options, apart from enforcing a minimum 5 bp overlap for pre-merger of overlapping paired-end reads (--peOverlapNbasesMin 5).

We performed structural annotation of the VIC030 and EYR054 soft-masked genome assemblies using the avian subset of the OrthoDB v10.1 protein database (Kriventseva et al. 2019), three additional avian proteomes from NCBI GenBank (*Chloebia gouldiae*, *Corapipo altera*, and *Neopelma chrysocephalum*; Supplementary Table 2), our genome-aligned coastal EYR RNA-seq reads, and AUGUSTUS-optimized training parameters. To do so, we employed the ‘--etpmode’ option of BRAKER2 v2.1.5 (Hoff et al. 2016; Hoff et al. 2019). We assessed the completeness of our annotations with BUSCO v3.0. Finally, we functionally annotated both genome assemblies using eggNOG-Mapper v2 (Huerta-Cepas et al. 2017) using the avian section of the eggNOG 5.0 orthology database as evidence (Huerta-Cepas et al. 2019).

### Identifying genomic scaffolds belonging to neo-sex chromosomes

To test H1, we assessed Z-, W-, or autosomal inheritance of scaffolds. For that, we mapped pools of WGS sequences from coastal EYR males and females to the coastal genome assembly, calculated mapping and kmer representation metrics of each sex, and summarized these differences in a Principal Component Analysis (PCA), which then was used to assign the inheritance to scaffolds according to their clustering patterns along PC1 (Supplementary Methods). We assessed the power of these sex-linked metrics to assign sex linkage to given genomic scaffolds by performing a linear discriminant analysis with cross-validation.

We determined whether sex-linked genomic scaffolds in the EYR054 and VIC030 genome assemblies mapped to the ancestral passerine sex chromosomes or autosomes by aligning each assembly separately against the female Zebra Finch (*Taenopygia guttata*) primary assembly (bTaeGut2.pri.cur.20181019) and mitochondrial (bTaeGut2.MT.20190308) genome assemblies (Rhie et al. 2020). We used the ‘asm20’ preset in minimap2 v2.14 (Li 2018) to allow better mapping of more diverged W-linked EYR scaffolds. For each genome scaffold, we filtered all alignment blocks by mapQ alignment score (>=40) and then sorted them by descending mapQ and block length, before designating the mapping target of the top hit as the main mapping target of the given scaffold. We considered sex-linked scaffolds that mapped to passerine autosomes to originate from a neo-sex chromosome in Eastern Yellow Robins.

### Assignment of sex linkage and mitochondrial function to annotated genes

By default, we allowed each annotated gene to inherit the sex linkage assignment of their parent genomic scaffold. However, sex linkage classification is most unambiguous in genomic regions that have undergone strong differentiation between neo-Z and neo-W. Longer neo-sex scaffolds contain lower proportions of differentiated sequence and are therefore more likely to be erroneously assigned as autosomal even when portions of these scaffolds may be detectably sex-linked. In our coastal assembly, this resulted in some genes on long scaffolds incorrectly being assigned as autosomal, although they belonged to a neo-sex chromosome. To rectify this, in EYR OGs with autosomal and W-linked sequences but no corresponding Z-linked sequence, we reassigned the autosomal transcript as the Z-linked gametolog. However, we did not reassign autosomal transcripts as W-linked ones when only sequences from Z-linked and autosomal scaffolds were present, instead considering them to not have been sequenced, for several reasons: (i) W-linked scaffolds are more easily distinguished from autosomal ones since they are only present in one sex, (ii) W-linked sequences should accumulate sex-linked signals faster than Z-linked chromosomes, because selection against deleterious mutations is weaker on hemizygous chromosomes, and (iii) W-linked scaffolds tend to be shorter than Z-linked and autosomal ones because repetitive regions on W-linked chromosomes prevent more contiguous assembly, so scaffold-length bias leading to incorrect sex-linkage assignment is less likely to affect W-linked than Z-linked or autosomal scaffolds. Finally, we classified passerine chr1A-derived genes as having mitochondrial or non-mitochondrial functions (N-mt or non-N-mt genes) according to the MitoMiner 4.0 database (Smith and Robinson 2009; Smith and Robinson 2018).

### Orthogroup construction and alignment processing for sex-linked EYR genes

To generate an appropriate dataset for testing H2 and H3, we first assigned annotated transcripts from sex-linked scaffolds in the VIC030 and EYR054 genome assemblies to orthogroups (OGs), defined as the sets of genes descended from a single gene in the last common ancestor of all the species being considered (Emms and Kelly 2019). To provide phylogenetic context for the evolution of EYR genes in each orthogroup, we retrieved proteomes and coding sequences (CDS) for twelve passerine species from GenBank (Supplementary Table 2) for use as outgroups, representing major clades of the core Passeroidea.

To build OGs, we extracted the longest transcript for each protein annotation from the VIC030 and EYR054 EYR genome assemblies and each outgroup proteome. We then generated orthogroups using OrthoFinder v.2.3.11 (Emms and Kelly 2019) by supplying the set of longest transcripts and an a priori species tree (Oliveros et al. 2019). We performed several steps of codon-aware alignment, sequence fragment rescue, and site filtering with the programs MACSEv2 (Ranwez et al. 2018), HMMCleaner (Di Franco et al. 2019), Gblocks (Talavera and Castresana 2007), as well as additional custom scripts, to arrive at a final dataset for use in downstream analyses (Supplementary Methods).

### Testing the effect of mitochondrial function on between-lineage differentiation of neo-sex gametologs

To test H2, we first calculated the coding sequence pairwise distances (we abbreviate this as Dxy, although it is usually applied to populations) of all sex-linked genes between the two lineages, partitioned by Z- or W-linkage, using the K80 model implemented in the ‘dist.dna’ function of the R package ape (Paradis et al. 2004); sites with gaps were deleted. Next, we modelled the crossed fixed effect of gene function (mitochondrial or non-mitochondrial function) and sex linkage (Z or W), which we determined to have a significant interaction, on between-lineage Dxy with a linear mixed-effects model (LMM), using the R package lme4 (Bates et al. 2015), including gene identity as a random effect. We calculated whether mitochondrial gene function had a significant association with the between-lineage Dxy distributions of Z- and W-linked genes separately.

We also tested whether amino acid substitution rates (dNdS) between EYR lineages were more often different for N-mt genes than for other (non-N-mt) sex-linked, Chr1A-derived genes. To do so, we first generated orthogroup-specific phylogenetic input trees for each orthogroup for use in all dNdS-based analyses with a custom script, following the species tree phylogeny in Oliveros et al. (2019) and including only the taxa present in each specific orthogroup. Sequences from each EYR lineage were allocated to groups depending on their assigned sex linkage, following the branch structure: (Outgroups, (Autosomal EYR, (W-linked EYR, Z-linked EYR))). We trimmed all codon sites with gaps in any EYR gametolog and then estimated gene-wide dNdS for each EYR neo-sex gametolog in a given OG, relative to the most appropriate available outgroup (Zebra Finch in ~96% of OGs), using CODONML from the package PAML v4.9 (Yang 2007). We calculated the difference in estimated dNdS of each inland-coastal pair of Z and/or W gametologs (dNdS(C-I)). We then tested the fixed effect of sex linkage and/or gene function on dNdS(C-I), which we first determined to not have a significant interaction, by fitting a linear mixed-effects model, using the R package lme4 (Bates et al. 2015) including gene identity as a random effect. We calculated whether gene function significantly predicts the magnitude of variation in dNdS between lineages.

### Influence of W- and Z-linkage on between-lineage differentiation of N-mt genes

To test H3, we used the linear models generated in the previous section, first testing the fixed effect of gene function (mitochondrial or not) and sex-linkage (Z or W) on between-lineage Dxy of N-mt and non-N-mt genes. We calculated whether there was a significant effect of W- or Z-linkage on the between-lineage Dxy distributions of N-mt and non-N-mt genes separately. Greater effect sizes of W linkage on between-lineage Dxy of N-mt genes relative to non-N-mt genes would be interpreted as a result of selection acting on mitonuclear interactions involving W-linked N-mt genes. We also used the linear model describing the effect of sex linkage and gene function on dNdS(C-I) of each sex-linked Chr1A-derived gametolog, calculating whether sex-linkage significantly predicts the magnitude of variation in dNdS between lineages. To visualize our dNdS-based results in the context of genome position, we performed a lift-over of genomic coordinates for all tested OGs to the female Zebra Finch genome (bTaeGut2) with Liftoff v1.6.1 (Shumate and Salzberg 2021) and generated a Manhattan plot with the R package ggplot2.

Finally, we sought individual N-mt gene candidates that could be involved in mitonuclear co-evolution by testing whether genes exhibited signals of positive selection. For each OG, we tested each sequence in a given phylogeny for positive selection with the adaptive Branch-Site Random Effects Likelihood model (aBSREL) implemented in HyPhy (Pond et al. 2005; Smith et al. 2015). The final aBSREL dataset was summarized with a Manhattan plot of branch p-values. Results of positive selection generated by the aBSREL model may be confounded by the presence of relaxed purifying selection, which can generate dNdS values over 1. We directly tested whether aBSREL-identified genes under putative positive selection were not false positives by testing them with the RELAX model, which tests for signals of intensified versus relaxed selection strength (Wertheim et al. 2015). To do so, we set all aBSREL-significant EYR branches as the foreground for the test. Genes with signals of positive selection in at least one lineage (aBSREL) as well as signals of intensified (rather than relaxed) selection (RELAX) were considered to be under differential positive selection between lineages. For both aBSREL and RELAX, nominal *p* values rarely survive multiple test correction because of the large number of tests conducted (Smith et al. 2015), so we used uncorrected p-values in our initial search for genes under positive selection, as is commonly practiced (Davies et al. 2020; Joseph et al. 2020; Schaschl and Wallner 2020).

We visualised the sequence alignments of identified N-mt genes under intensified positive selection using the software Geneious Prime 2021.0.3 (https://www.geneious.com), to assess intactness of gene structure and identify features that might indicate changes to function. We assessed whether any exon structures missing in genes from one lineage were not merely the artefactual result of incorrect annotation. To do so, we cross-mapped the homologous exon from the other lineage, if present, onto the genome assembly of the first using BLASTN 2.2.31+, and then assessed the likelihood that found exon-like sequences could be translated together with annotated exons.

## Results

### Coastal genome assembly and annotation completeness

**Fig. 2:**
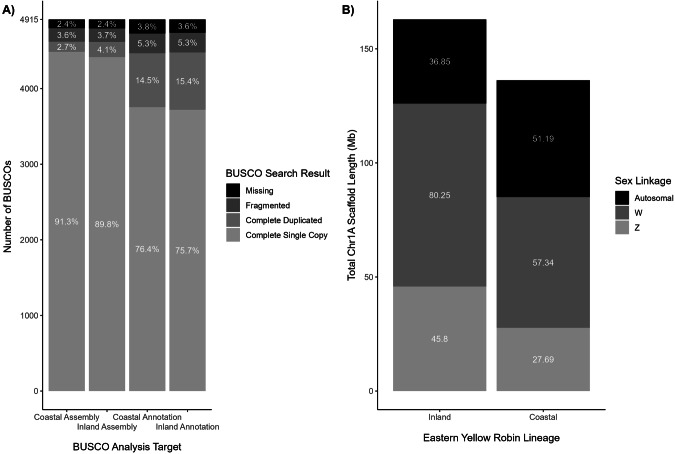
Two assessed metrics for the inland (EYR054) and coastal (VIC030) genome assemblies and annotations. **A** Percentages on the plot indicate the proportion of chicken-derived Benchmarking Universal Single-Copy Orthologs (BUSCOs) that were recovered in each given category. A large number of BUSCOs were annotated as duplicates in each genome, likely the result of BUSCOs (normally autosomal) that have diverged into sex-linked variants in EYR. **B** Total length (Mb) of inferred sex-linked and autosomal Zebra Finch (ZF) chromosome 1A-mapping scaffolds for the inland and coastal genome assemblies.

We obtained ~205 Gb ( ~ 170x coverage) of Illumina short-read data, ~2.3 Gb of sequencing data in 326,027 Nanopore long reads (*N*_50_ = 12,839 bp), and a further ~13.0 Gb (~10x coverage) of sequencing data in 1,554,012 PacBio long reads (*N*_50_ = 11,583 bp), after quality filters, from a coastal EYR (VIC030). We assembled this individual’s genome to a total length of 1.199 Gb, when considering only contigs >= 1 kbp in length (Supplementary Table 3). A scaffold N50 length of this new assembly was 1.94 Mb, twice that of the published inland EYR genome assembly (Assembly length = 1.228 Gb, N50 = 0.99 Mb; Gan et al. (2019)). We recovered 94.0% of avian BUSCOs as complete single copies in the new assembly (Fig. 2A). BUSCO assessment of our genome annotations (predicted proteomes) recovered 76.4% and 75.7% of single-copy chicken orthologs as single copies in the inland and coastal EYR assembly annotations respectively, as well as a further 15.4% and 14.5% as duplicates respectively (Fig. 2A). The increase in BUSCO duplication rates found in our annotation over the assembly is most likely due to the presence of sex-linked variants derived from Chr1A BUSCOs that were identified by the annotation process.

### Neo-sex chromosome identification

**Fig. 3:**
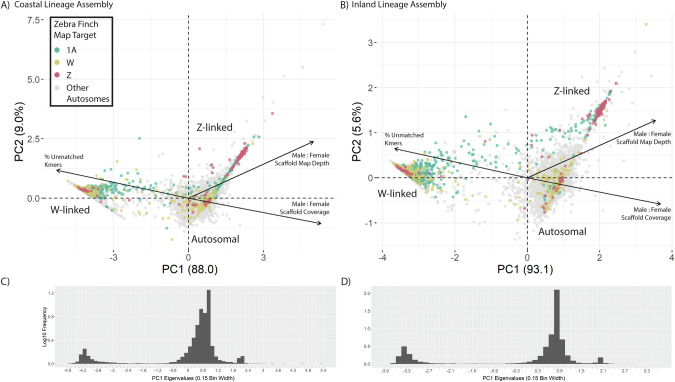
Sex-linkage discrimination of all genomic scaffolds in both Eastern Yellow Robin assemblies used in this study. Principal component analysis of three scaffold metrics for (**A**) coastal and (**B**) inland genome assemblies respectively (proportion of unmatched single-copy kmers, male-to-female ratio of mean scaffold mapped read depth, and male-to-female ratio of horizontal scaffold mapping coverage by WGS reads). Each dot in the plot represents one genomic scaffold, and colours correspond to the best map targets in the female Zebra Finch assembly bTaeGut2 (Teal: Chr1A; Beige: ChrW; Red: ChrZ; Grey: autosomes other than Chr1A). **C**, **D** Distribution of scaffolds along PC1 in each EYR assembly. Eigenvalues in histogram are aligned and correspond with respective PCAs above. Scaffolds in both lineages’ genome assemblies sort into a trimodal distribution along PC1.

Our analysis of sex-linkage strongly suggests that the coastal lineage of Eastern Yellow Robins possesses a neo-sex chromosome system that is homologous to the inland lineage. Using PC1 eigenvalues from the PCA of scaffold sex-linkage metrics, we found neo-sex chromosomes in our coastal EYR assembly that mapped to Zebra Finch chromosome 1A, similar to those in the inland assembly (Gan et al. (2019) and re-analysis here). Z-linked, W-linked, and autosomal scaffolds generally clustered separately on PC1 vs PC2, with PC1 explaining the majority of variation in the dataset (88% and 93.1% of variance for the coastal and inland assemblies respectively, Fig. 3). Linear discriminant analysis with cross-validation showed strong prediction accuracy with scaffold sex-linked metrics (98.89% accuracy when predicting all scaffolds’ sex linkages or 95.65% when applied only to scaffolds with final sex-linked chromosomes assignment post-hoc; Supplementary Table 4). The total length of identified neo-sex scaffolds was lower for the coastal lineage than for the inland one (Fig. 2B), likely owing to apparent under-assignment of scaffolds to neo-Z or neo-W in the more contiguous coastal assembly. It is highly unlikely that the coastal EYR lineage actually possesses shorter neo-sex chromosomes than the inland lineage, as this would imply either an independent neo-sex transition of ancestral chr1A or highly elevated rates of chromosomal degradation relative to the inland lineage.

### Patterns of between-lineage differentiation in neo-sex genes

**Fig. 4:**
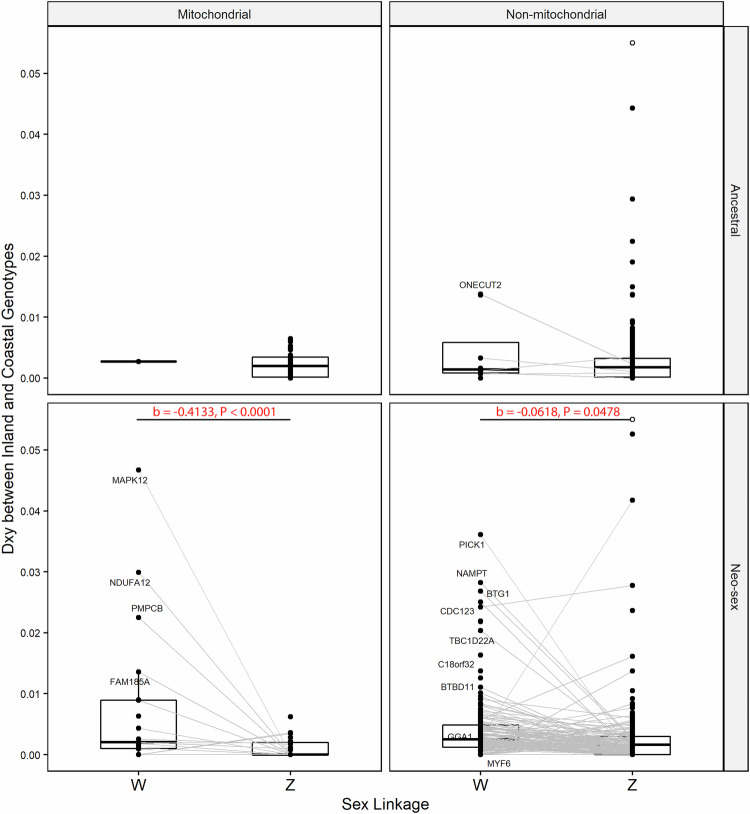
Distribution of sequence divergence (Dxy) values calculated between inland and coastal EYR lineages. Genes were partitioned by mitochondrial vs non-mitochondrial gene function (left and right facets), ancestral/neo-sex genomic location (top and bottom facets), and W versus Z sex linkage (left and right within facets). Genes with Z- and W-linked gametologs represented in both lineages are connected by grey lines. Genes with Dxy values outside the plot scale limits are depicted with open circles (untruncated scale in Supplementary Fig. 3). Genes are labelled if the difference in between-lineage Dxy (ΔDxy) of their Z- and/or W-linked gametologs are significant outliers (Z-score >1.96), relative to the overall distribution of ΔDxy.

**Fig. 5:**
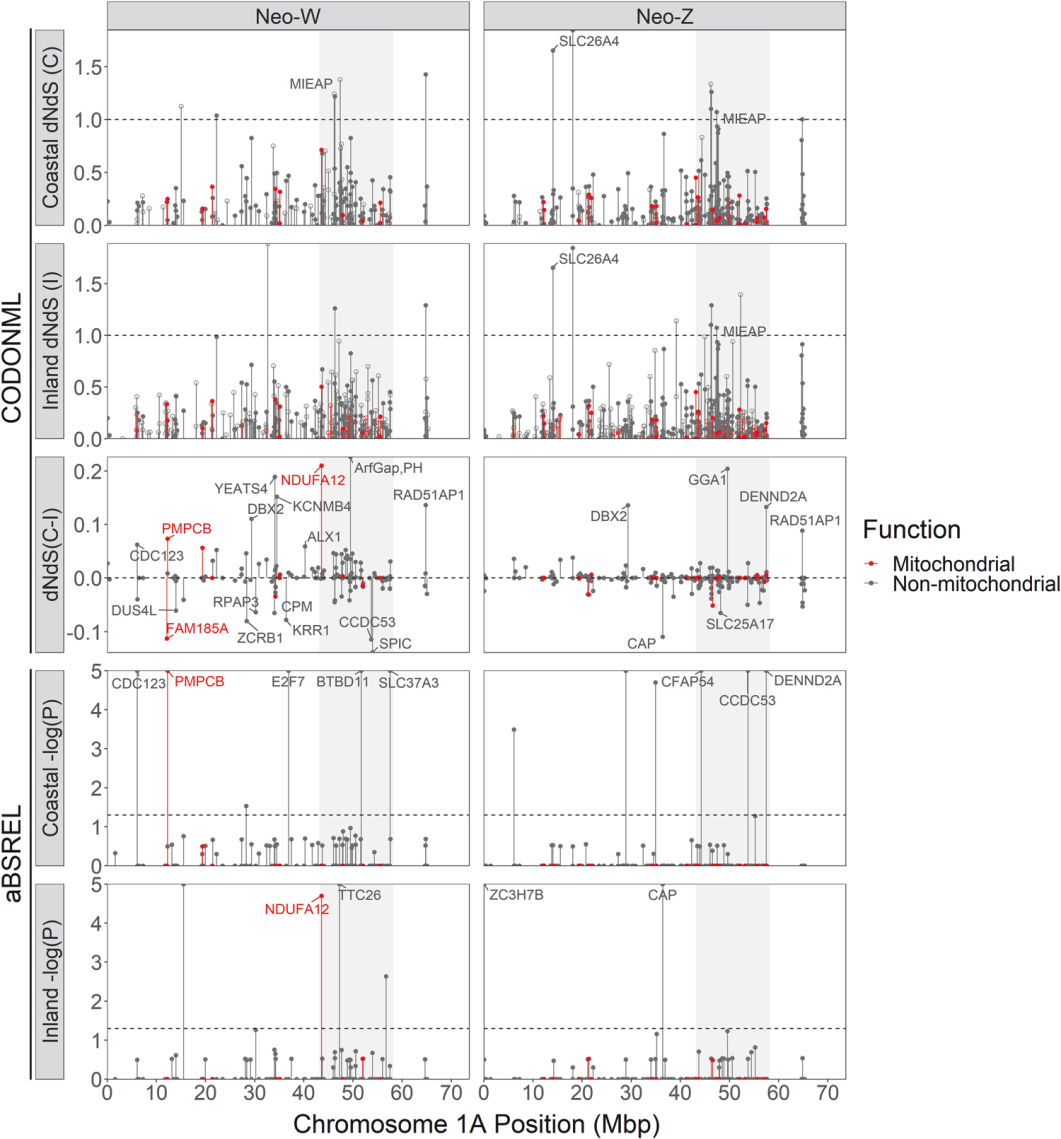
Manhattan plots of N-mt and non-N-mt genes (red and black respectively) located on neo-W and neo-Z chromosomes (left and right columns). Plots depict CODONML gene-wide dNdS estimates (top two rows), differences in CODONML dNdS rate estimates between EYR lineages (middle row, dNdS(C-I)), and –log(P) values for aBSREL tests for positive selection for the coastal and inland EYR lineages (bottom two rows). Zebra Finch chromosome 1A is used to provide reference position genome coordinates for each gene and the region of high between-lineage nuclear differentiation previously found in Morales et al. (2018) is indicated by the greyed region. Only gametologs that exhibit significant signals of positive selection intensification rather than relaxed purifying selection are labelled in all plots except the row depicting dNdS(C-I) (also see Supplementary Table 6 and Supplementary Fig. 4). For the row depicting dNdS(C-I), genes above the horizontal grey dotted line experience higher protein evolution in the coastal EYR lineage, and ones below the line higher in the inland lineage, with 1% outliers named.

There was no significant difference in the between-lineage sequence divergence (Dxy) between genes with- and without mitochondrial function when comparing neo-W and neo-Z gametologs separately (LMM; *P* > 0.05 for both sets of comparisons; Fig. 4 & Supplementary Fig. 2). There were also no N-mt genes with dNdS > 1, indicating a lack of evidence for broad patterns of positive selection on neo-sex N-mt genes; several non-N-mt genes had dNdS > 1, but this class was considerably more numerous overall (Fig. 5). Between-lineage sequence divergence (Dxy) in neo-W N-mt genes (Dxy statistics: Q1 = 0.001, Q2 = 0.002, Q3 = 0.0089, max = 0.0467) was significantly higher than in their respective neo-Z N-mt gametologs (Dxy statistics: Q1 = 0, Q2 = 0, Q3 = 0.002, max = 0.0062) (b = −0.413, *P* < 0.0001, Fig. 4). This effect in N-mt genes was ~7 times higher than in non-N-mt genes (b = −0.062, *P* = 0.048, Fig. 4).

Four neo-W N-mt genes (Z-score > 1.96) had significantly elevated sequence divergence between lineages relative to their neo-Z counterparts (bottom panels; Fig. 4): NADH Dehydrogenase [Ubiquinone] 1 Alpha Subcomplex Subunit 12 (NDUFA12), Mitochondrial-Processing Peptidase Subunit Beta (PMPCB), Family with Sequence Similarity 185 Member A (FAM185A), and Mitogen-Activated Protein Kinase 12 (MAPK12). By contrast, we did not identify any N-mt genes on neo-Z with significantly elevated Dxy between lineages relative to their neo-W counterparts. The dNdS ratios of W-linked gametologs in each lineage were different from one another significantly more often than those of their Z-linked gametologs (CODONML, dNdS(C-I), Fig. 5; b = −0.261, *P* = 0.02), an indication that W-linked gametologs evolved more independently between lineages than did Z-linked gametologs. Gene function (N-mt vs non-N-mt) was not a significant predictor of whether genes had different functional evolution rates between lineages (LMM; b = −0.033, *P* = 0.88). In total, we tested 953 genes represented in both EYR lineages across 792 sex-linked OGs for positive selection in EYR branches with the aBSREL model (Supplementary Table 5).

Two W-linked N-mt genes exhibited signals of intensification of positive selection rather than relaxed purifying selection: NDUFA12 in the inland lineage, and PMPCB in the coastal one (aBSREL, Fig. 5; RELAX, Supplementary Table 6). These two genes were also identified as Dxy outliers (Fig. 4). A third W-linked Dxy outlier, FAM185A, did not show significant signal of positive selection, and the fourth Dxy outlier, MAPK12, could not be ruled out as being under relaxed purifying selection rather than intensified positive selection (RELAX; Supplementary Table 6).

**Fig. 6:**
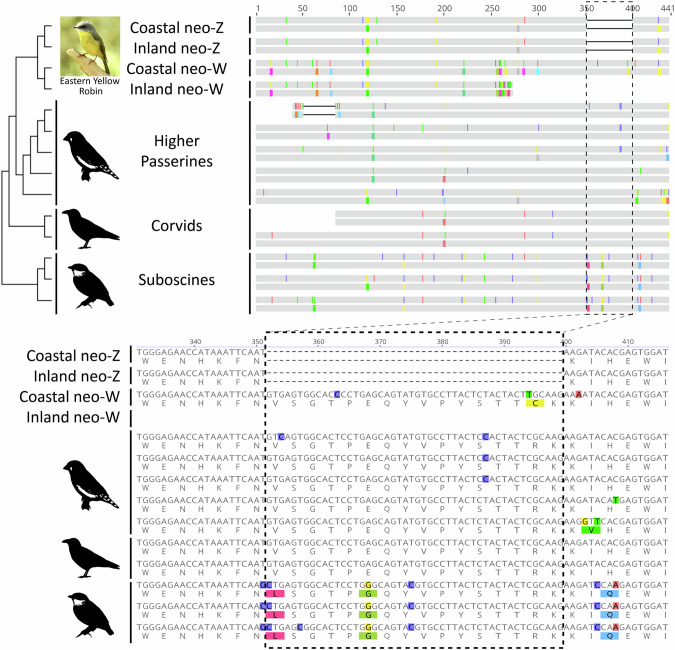
Trimmed 441 bp coding sequence alignment of the NDUFA12 orthogroup. (Top) Each double row depicts the nucleotides above and the corresponding amino acid translations below, with substituted positions coloured. A cladogram of taxa included in the orthogroup is placed on the left. The inland neo-W EYR gametolog appears to have lost exon 4. (Bottom) A closer view of the NDUFA12 alignment reveals a 16-codon gap in neo-Z gametologs in both EYR lineages, whereas the coastal (but not inland) neo-W EYR gametolog possesses this region.

Inspection of NDUFA12, a gene usually encoded as four exons within ~10 kbp of each other in vertebrates, revealed a truncation in the inland neo-W gametolog, including the complete loss of the canonical exon 4 (273 bp, 61.9% of the total coding sequence length) and the stop codon (Fig. 6). Cross-mapping of the coastal neo-W NDUFA12 exon 4 sequence to the inland genome assembly generated no hits within the ~126.4 kbp remaining on the scaffold containing the first three exons (QKXG01000310.1, 206,092 bp long). Instead, an exon 4-like sequence was found on a different genomic scaffold (QKXG01009618.1, 29,575 bp long) starting at the 7419 bp position. Similarly, inspection of the sequence alignments of PMPCB, FAM185A, and MAPK12 showed that the neo-W gametologs of both lineages also experienced significant deletions (~50–60% of total sequence alignment length), although the neo-Z gametologs remain intact. We therefore infer that the dNdS-based aBSREL and RELAX tests likely falsely detected a signal of intensified positive selection in the coastal neo-W genotypes of NDUFA12 and PMPCB.

## Discussion

### Complementary EYR genome assemblies provide powerful resources for studying genes undergoing incipient sex- and environment-linked evolution on neo-sex chromosomes

The majority of examples in which mitonuclear interactions involve sex-linked nuclear partners have been in systems with gene candidates on old or diploid sex chromosomes (X or Z) (Haddad et al. 2018; Lopez et al. 2021; Trier et al. 2014), with few exceptions (Wang et al. 2022). Our work presents a rare empirical indication that complete co-inheritance of W chromosomes with mtDNA could present an opportunity for rapid co-evolution of mitonuclear genotypic combinations. This phenomenon has previously been debated (Hill 2014; Irwin 2018; Smeds et al. 2015), but appropriate evidence has been lacking given the previous availability of only old sex chromosomes with few if any mitonuclear genes (Dean et al. 2014). Any W-linked selection on mitonuclear interactions likely occurs only before the genes involved have been degraded to the point of loss of function (Berlin and Ellegren 2006; Berlin et al. 2007). Our study illustrates how neo-sex chromosome systems can be used to study the dynamics of sex-linked mitonuclear co-evolution, which could be common but transient in climate-driven adaptation and speciation. This points towards a need for more studies involving independent neo-sex chromosome systems exploring sex-linked differentiation of N-mt genes in response to environment-driven selection.

The coastal EYR genome assembly released in this study directly complements the previously published inland EYR assembly (Gan et al. 2019) and enables detailed study of sex- and environment-linked selection on neo-sex genes. Chromosome 1A is one of the largest avian chromosomes and correspondingly bears a large number of genes, including the largest concentration of N-mt genes known in avian genomes (Morales et al. 2018). The data we have presented here demonstrate that Eastern Yellow Robins offer a so-far unique opportunity to test hypotheses concerned with the evolutionary and functional consequences of sex-linkage of large numbers of N-mt genes. By first showing that both EYR lineages possess homologous neo-sex chromosomes, we can subsequently explore whether observed divergence in newly sex-linked genes between lineages is associated with sex- or environment-linked selection, rather than whether a given homologue is sex-linked in one lineage or not. We were therefore able to test a large number of annotated neo-sex N-mt genes (accounting for nearly 10% of 792 total annotated genes) and non-N-mt comparators in our searches for between-lineage differentiation among neo-sex genes. More specifically, 33 neo-W N-mt, 35 neo-Z N-mt, >330 neo-W non-N-mt, and >286 neo-Z non-N-mt genes were tested.

Avian neo-sex chromosome transitions were first discovered in passerines of the superfamily Sylvioidea, and avian neo-sex evolutionary studies have thus far mainly focused on members of this diverse clade. These report a growing list of chromosomes that have fused with Z or W (including chromosomes 3, 4A, 5, and 8), but so far this does not include Chr1A (Pala et al. 2012a; Pala et al. 2012b; Sigeman et al. 2020; Sigeman et al. 2022). The EYR system therefore provides a taxonomically and chromosomally independent model in which to study the evolution of neo-sex chromosomes and their genes.

### Differences in coding sequence evolution on neo-Z and neo-W chromosomes

Strong differentiation in mitogenomes between climate-associated lineages of EYR leads to the expectation that one or more co-adapted N-mt genes will also be strongly differentiated (Hill 2014; Sloan et al. 2017). On average, we did not find that N-mt genes were more diverged between lineages compared to non-N-mt genes on the same neo-sex chromosome, although average effects may not necessarily be expected: Hill et al. (2019) note that ‘mitonuclear incompatibilities can be caused by a small number of variants that need not change amino acid sequence and that may not be proportional to overall sequence divergence’. Nonetheless, we did find evidence of selective pressures acting differently on the set of N-mt genes located on neo-Z and their neo-W counterparts. Neo-Z N-mt genes were nearly undifferentiated between coastal and inland lineages, consistent with strong purifying selection and male-mediated gene flow between lineages, whereas neo-W N-mt genes were relatively more differentiated between coastal and inland lineages. Neutral drift should contribute to between-lineage differentiation of N-mt and non-N-mt genes on W-linked chromosomes equally, so we consider the excess in between-lineage differentiation of neo-W N-mt genes relative to their neo-Z gametologs (which is ~7 times larger than the equivalent increase in neo-W versus neo-Z gametologs of non-N-mt genes) to be reflective of accelerated differentiation stemming from co-evolution with mitogenomes.

Genes located on Z chromosomes are thought to evolve faster than those on autosomes, an effect that might be amplified in the case of N-mt genes because their inheritance is partially decoupled from the matrilineal mitochondrion, which would reduce the effectiveness of linked selection on these two evolutionary units, allowing them to evolve more independently of each other (Hill 2014; Irwin 2018). Because genes on Z-linked chromosomes spend 67% of their time in males and experience accelerated evolutionary rates because of the faster-Z effect, Z-linkage has been thought to promote observable acceleration of differentiation under selection (Barton and Charlesworth 1998; Hill 2014; Mank et al. 2007). However, our results do not show these patterns. Instead, low differentiation of neo-Z N-mt gametologs is consistent with strong purifying or similar directional selection and/or a homogenizing effect of previously-demonstrated male-mediated nuclear gene flow between coastal and inland lineages (Morales et al. 2017; Pavlova et al. 2013). This suggests that phenotypes encoded by neo-Z N-mt genes are exposed to selection from both inland and coastal environments and could be intermediately adapted to both, and/or are subject to little selection. At the same time, lineage-specific differentiation of neo-W N-mt genes that does arise could be subsequently maintained by their complete co-association with the respective mtDNA genotype, as has been previously proposed to occur (Berlin et al. 2007; Irwin, 2018; Pavlova et al. 2013).

### Evolution of sex-linked NADH dehydrogenase [Ubiquinone] 1 alpha subcomplex subunit 12 (NDUFA12) in Eastern Yellow Robins

One of the five N-mt genes previously proposed to be candidate drivers of between-lineage differentiation in EYR (Morales et al. 2018), NADH Dehydrogenase [Ubiquinone] 1 Alpha Subcomplex Subunit 12 (NDUFA12), is here independently identified as a promising candidate driver of mitonuclear incompatibilities in this study. NDUFA12 is an accessory subunit of OXPHOS Complex I and is likely involved in stabilising the formation of the matrix arm of Complex I during assembly (Rak and Rustin 2014; Ton et al. 1997). NDUFA12 impairment is strongly associated with Leigh syndrome in humans, a paediatric mitochondrial disorder characterized by brain-specific anomalies and early death (Magrinelli et al. 2022; Ostergaard et al. 2011; Rak and Rustin 2014; Torraco et al. 2021) for which no fully compensatory mechanisms are known (Adjobo-Hermans et al. 2020; van de Wal et al. 2022).

In EYR, neo-Z NDUFA12 gametologs are present and undifferentiated between lineages, while neo-W NDUFA12 gametologs are differentiated between lineages and also from their neo-Z gametologs. The inland neo-W gametolog of NDUFA12 appears to have lost exon 4, with no homologous sequence present on the remainder of the genomic scaffold containing the first three exons. We consider it unlikely that a separate exon 4-like sequence found on another genomic scaffold could rescue the gene as it would be separated from exon 3 by at least 130 kbp, within which another gene, ALX1, would be nested (from the 145,584–169,494 bp positions on scaffold QKXG01000310.1). Additionally, the coastal neo-W gametolog retains a 16-codon section that is present in all other passerines tested, but absent in the neo-Z gametologs of either EYR lineage. It is very unlikely for two independent high-coverage genome assemblies to erroneously show the same deletion as a matter of random chance, so we are confident that this unprecedented deletion in neo-Z NDUFA12 is real. Furthermore, given that W-linkage should expose genes to higher levels of drift and degradation compared to Z-linkage, it also seems unlikely that the coastal W-linked gametolog retained this segment by chance, suggesting that its retention may have been selected for. Overall, this is consistent with a hypothesis of female-biased selection on the co-evolution of this gene with the mitogenome in the coastal lineage of EYR. It is surprising that neo-Z NDUFA12 gametologs in Eastern Yellow Robins have a deletion not seen in all other passerines tested, and is likely to have resulted in functional changes that will need to be characterized in order to understand their relative impact on males and females living in different environments.

Differentiated neo-W NDUFA12 gametologs may contribute towards gene flow barriers between coastal and inland lineages. Under the hypothesis that differentiated neo-W gametologs and the mitogenome in each lineage interact to confer females a selective advantage in their respective environments, hybrid females should be better adapted to their mothers’ home environments because they inherit a co-adapted mitonuclear set of neo-W gametologs and mitogenome from them. Hybrid females dispersing from hybrid zones of intermediate environments towards their father’s home environments should therefore experience reduced fitness and contribute less towards cross-lineage gene flow. Conversely, hybrid males should be more poorly adapted to their fathers’ home environments because even though they possess the same neo-Z NDUFA12 gametologs as non-hybrid males, they inherit mtDNA adapted to the opposite environment from their mothers. The expected overall effect of these dynamics is to bias gene flow between lineages towards male-mediation even though Eastern Yellow Robin males exhibit natal philopatry, which is in good agreement with previous observations (Morales et al. 2017; Pavlova et al. 2013).

### Concluding remarks

The Eastern Yellow Robin is a striking example of a system exhibiting environment-associated mitolineages patterning that strongly contrasts with differentiation in the majority of the nuclear genome (Morales et al. 2017; Pavlova et al. 2013). This pattern of differentiation has been posited to be explained by female-linked selection and/or incompatibilities against a background of male-mediated gene flow, and nuclear genes involved in mitonuclear interactions have been repeatedly implicated by several studies (Morales et al. 2015; Morales et al. 2017; Morales et al. 2018). Our study provides the first direct evidence concerning the extent of differentiation of neo-W- and neo-Z-linked nuclear N-mt genes between individuals from two environment-associated lineages of the Eastern Yellow Robin. Our identification of one N-mt gene, NDUFA12, as a candidate driver of mitonuclear incompatibility and/or positive selection involved in lineage differentiation prompts further characterization of the functionality and allelic frequencies of the NDUFA12 gametologs annotated in both EYR lineages, to enhance understanding of whether and how it enables adaptation to different climates.

The Anthropocene has been characterized by unprecedented levels of biodiversity decline and environmental changes. Rapid environmental and climatic changes can generate strong selective pressures on lineages living in affected areas. Our work on an early-branching passerine reveals that neo-sex chromosome systems containing functional genes may present additional opportunities for climate-driven selection. Furthermore, in ZW sex-determined species, our results indicate that the W chromosome may play a much larger role in the early steps of environment-driven speciation than previously anticipated, mediated by mitonuclear interactions.

## Supplementary information

S1. Supplementary Figures and Tables

S2. Supplementary Methods

## Data Availability

All accession numbers for sequencing data are listed in the Supplementary Information. Coding scripts and other data are available at https://bridges.monash.edu/articles/dataset/Neo-sex_chromosome-encoded_mitochondrial_genes_in_Eastern_Yellow_Robin_lineages/24531070.
