# Supplementary material for: Accelerated differentiation of neo-W nuclear-encoded mitochondrial genes between two climate-associated bird lineages signals potential co-evolution with mitogenomes
Source: Heredity (Edinb). 2024 Aug 22;133(5):342–54. doi: 10.1038/s41437-024-00718-w (PMC11527876; doi:10.1038/s41437-024-00718-w)
Supplement: Supplementary file 1 — S1. Supplementary Figures and Tables [file 41437_2024_718_MOESM1_ESM.docx]

**Supplementary material for:**

**Accelerated differentiation of neo-W nuclear-encoded mitochondrial genes between two climate-associated bird lineages signals potential co-evolution with mitogenomes**

Gabriel Weijie Low, Alexandra Pavlova, Han Ming Gan, Meng-Ching Ko, Keren R Sadanandan, Yin Peng Lee, Nevil Amos, Lana Austin, Stephanie Falk, Damian Dowling, Paul Sunnucks

### Supplementary Table 1. Sample information for individuals used in deep sequencing for genome assembly and whole-genome resequencing for sex-linked scaffold metric calculations.


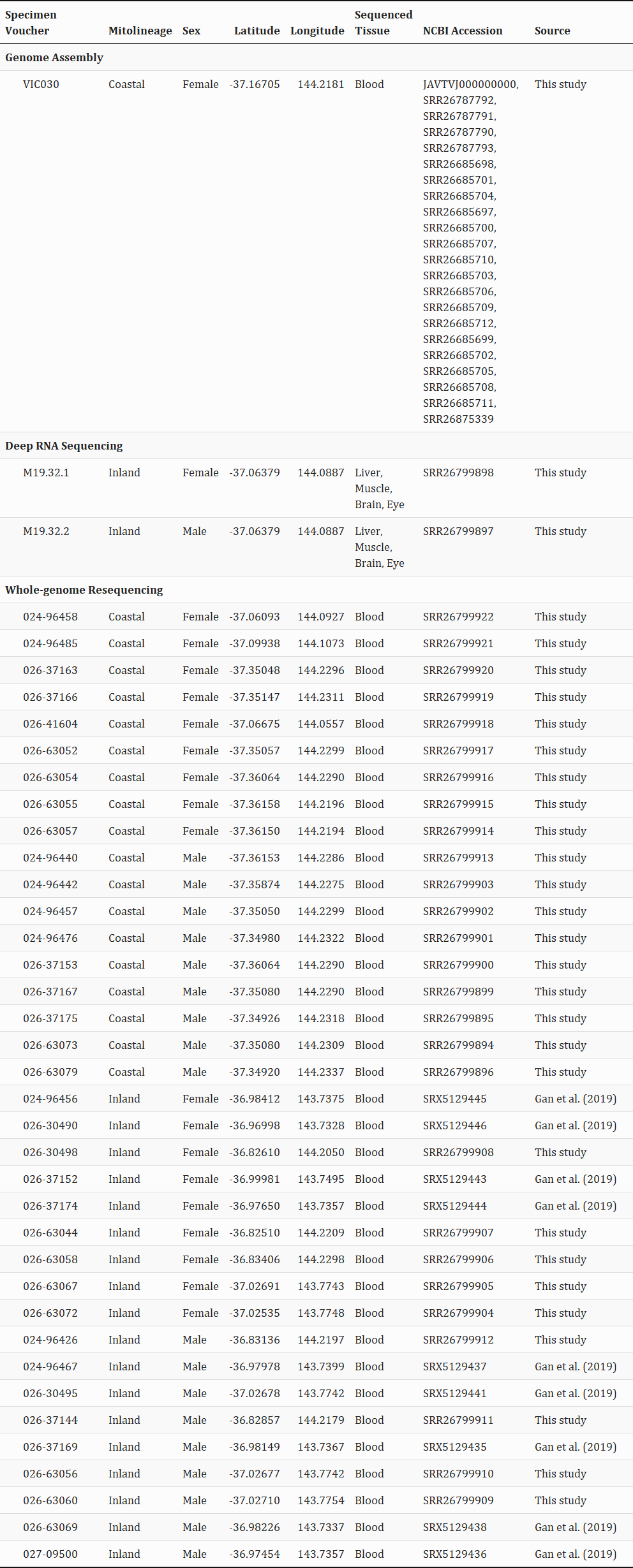


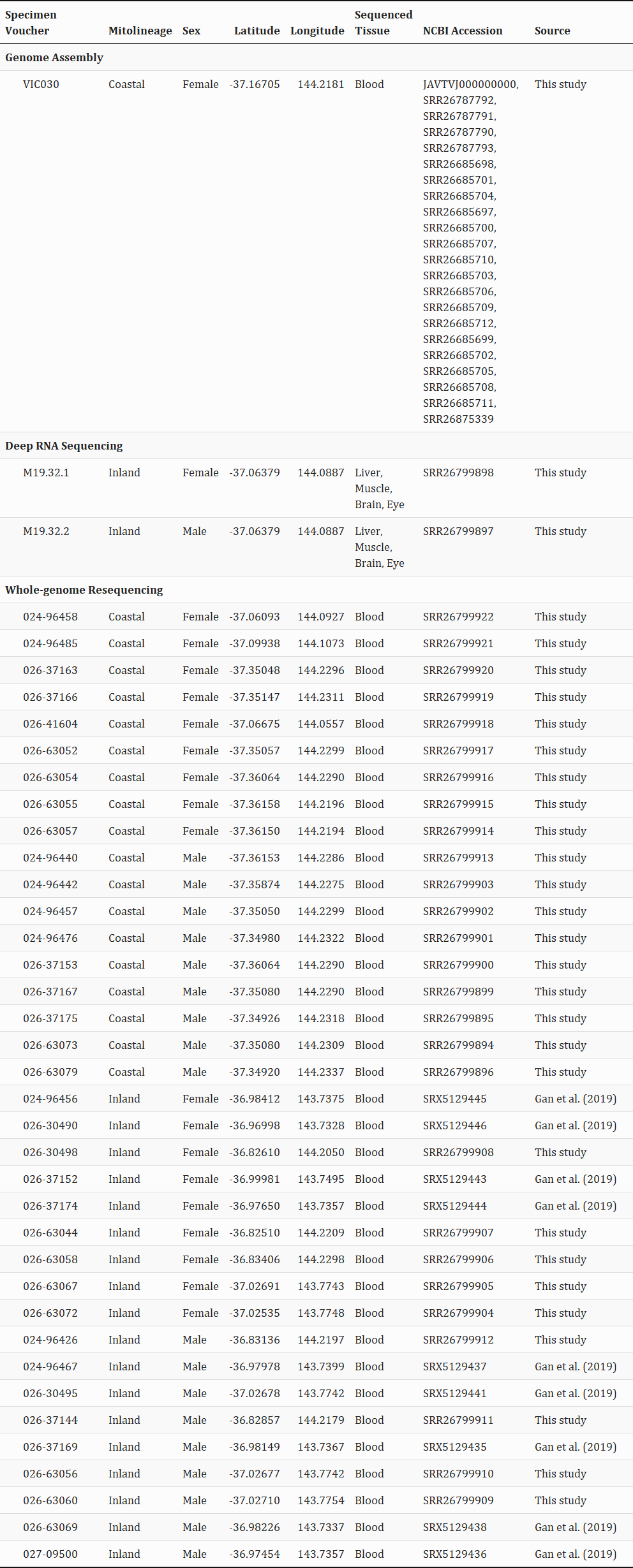


### Supplementary Table 2. Accession numbers for outgroup genomes and proteomes used in analyses.


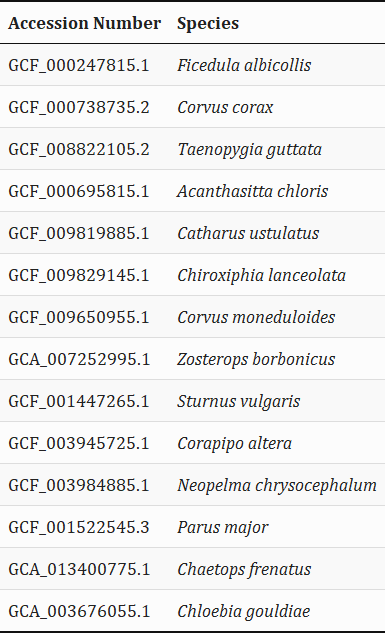


### Supplementary Table 3. Coastal Eastern Yellow Robin genome assembly (specimen voucher VIC030) metrics generated by QUAST (Gurevich *et al*, 2013).


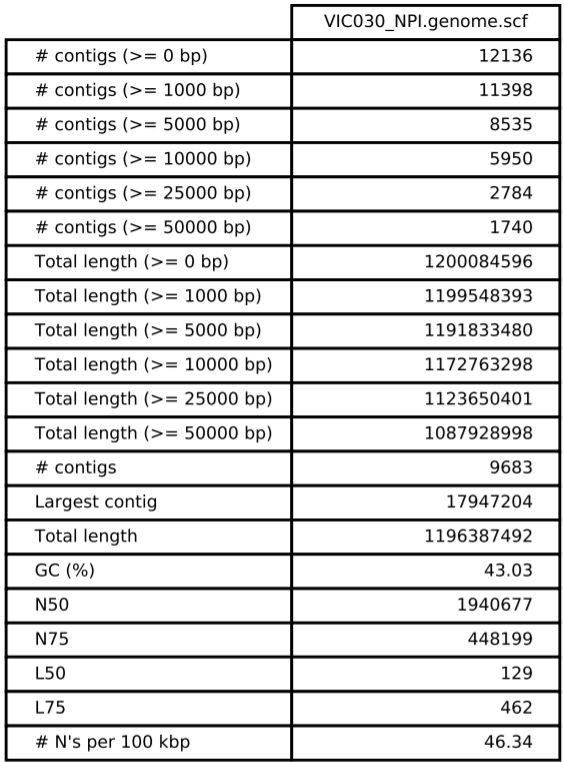


### Supplementary Table 4. Confusion matrices and statistics output of linear discriminant analysis on sex-linked metrics used to assign sex linkage to the global set of VIC030 genomic scaffolds (top) or the subset that was assigned to be sex-linked by our PCA-based approach (bottom).

Global VIC030 genomic scaffold set:

Confusion Matrix and Statistics

Reference

Prediction auto W Z

auto 6693 52 16

W 0 1139 0

Z 23 1 378

Overall Statistics

Accuracy : 0.9889

95% CI : (0.9864, 0.9911)

No Information Rate : 0.809

P-Value [Acc > NIR] : < 2.2e-16

Kappa : 0.9653

Mcnemar's Test P-Value : 9.893e-12

Statistics by Class:

Class: auto Class: W Class: Z

Sensitivity 0.9966 0.9555 0.95939

Specificity 0.9571 1.0000 0.99697

Pos Pred Value 0.9899 1.0000 0.94030

Neg Pred Value 0.9851 0.9926 0.99797

Prevalence 0.8090 0.1436 0.04746

Detection Rate 0.8062 0.1372 0.04553

Detection Prevalence 0.8144 0.1372 0.04842

Balanced Accuracy 0.9769 0.9778 0.97818

PCA-assigned sex-linked genomic scaffolds only:

Confusion Matrix and Statistics

Reference

Prediction W Z auto

W 1139 0 0

Z 1 378 0

auto 52 16 0

Overall Statistics

Accuracy : 0.9565

95% CI : (0.9453, 0.966)

No Information Rate : 0.7516

P-Value [Acc > NIR] : < 2.2e-16

Kappa : 0.8915

Mcnemar's Test P-Value : 6.988e-15

Statistics by Class:

Class: W Class: Z Class: auto

Sensitivity 0.9555 0.9594 NA

Specificity 1.0000 0.9992 0.95712

Pos Pred Value 1.0000 0.9974 NA

Neg Pred Value 0.8814 0.9867 NA

Prevalence 0.7516 0.2484 0.00000

Detection Rate 0.7182 0.2383 0.00000

Detection Prevalence 0.7182 0.2390 0.04288

Balanced Accuracy 0.9778 0.9793 NA

### Supplementary Table 5. Number of N-mt and non-N-mt orthogroups for which pairwise sequence divergence (Dxy) relative to orthogroup outgroups, Dxy between EYR lineages, Dxy between ZW gametologs were calculated, or included in aBSREL tests for positive selection. Gene function was classified according to the MitoMiner 4.0 database (Smith and Robinson, 2018), which includes predicted genes with mitochondrial function.


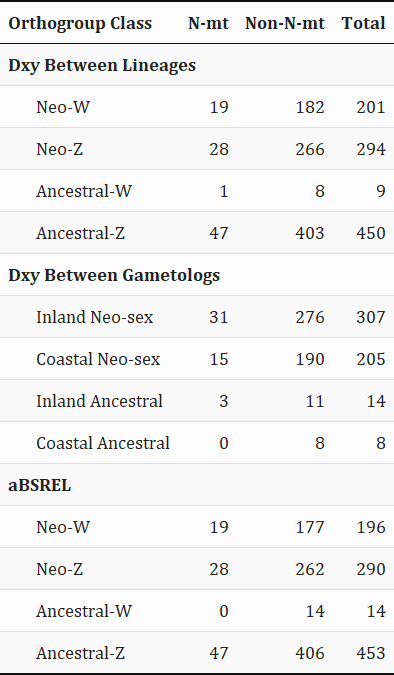


### Supplementary Table 6. All genes exhibiting putative positive selection during aBSREL (Smith *et al*, 2015) and CODEML (Yang, 2007) tests were tested for selection intensification or relaxation with RELAX (Wertheim *et al*, 2015). Calculated P-values refer to the likelihood that tested genes experience selection intensification or relaxation (Test-wide K > 1 or < 1 respectively) relative to the background of all other branches in a given orthogroup. Reported branch-specific relaxation parameters (branch-specific K) give an indication of the variation of selection intensity along EYR branches. Only orthologs exhibiting significant signals of selection intensification were labelled in main Figure 4.


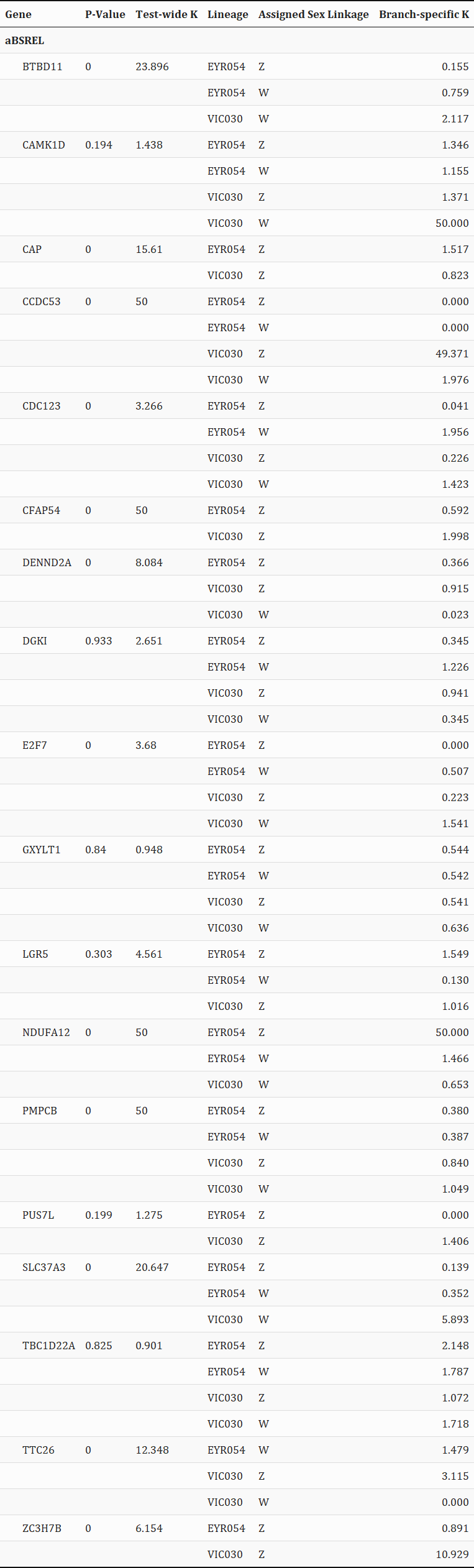


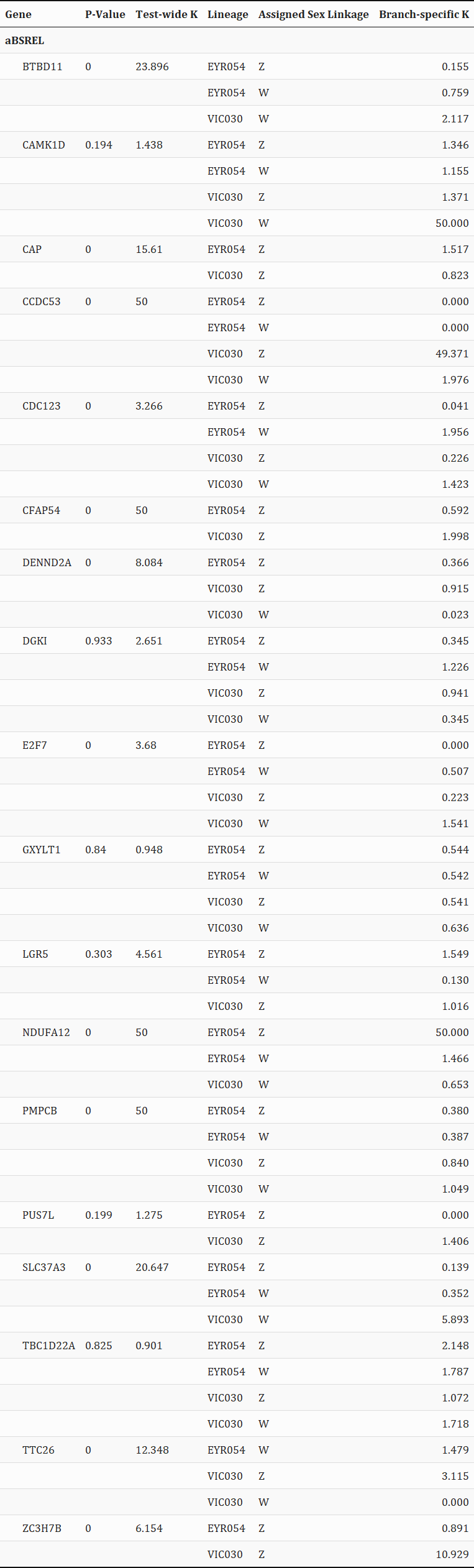


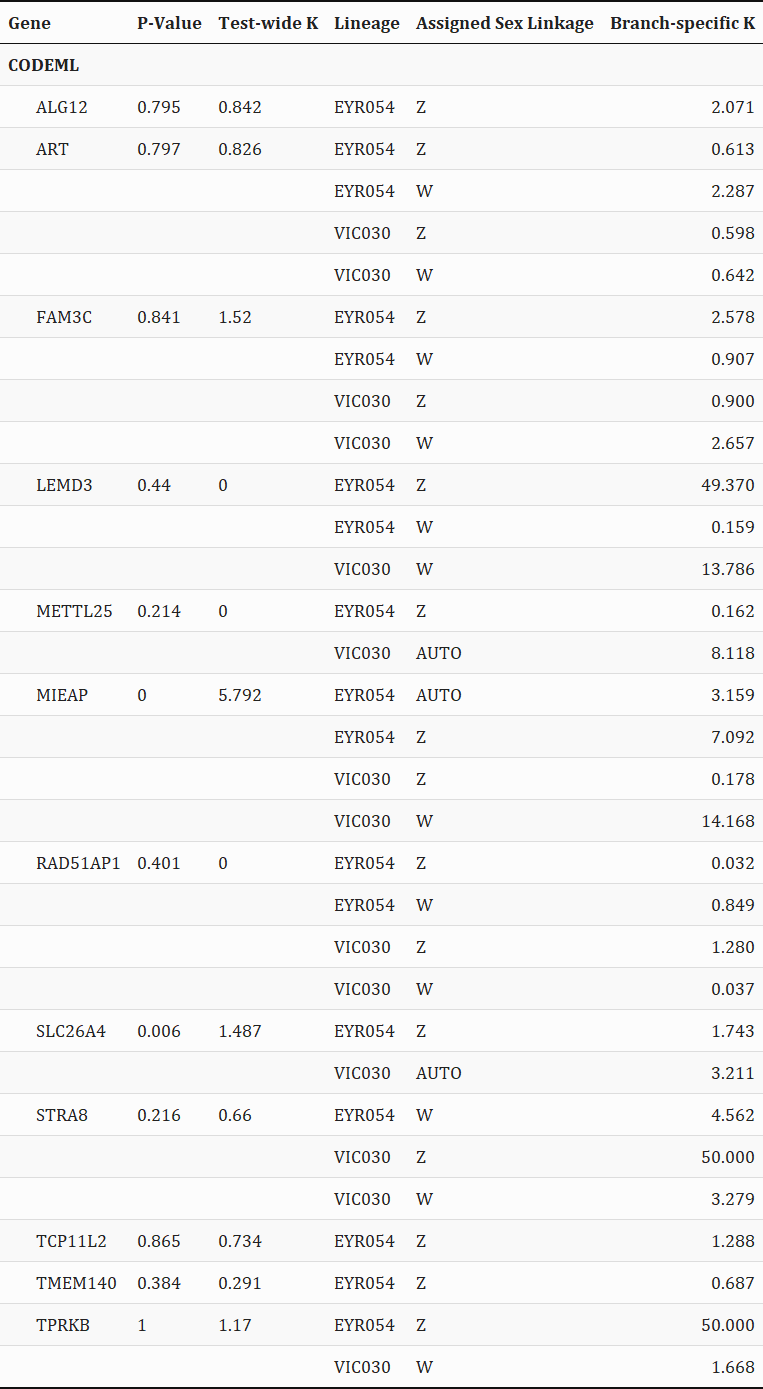


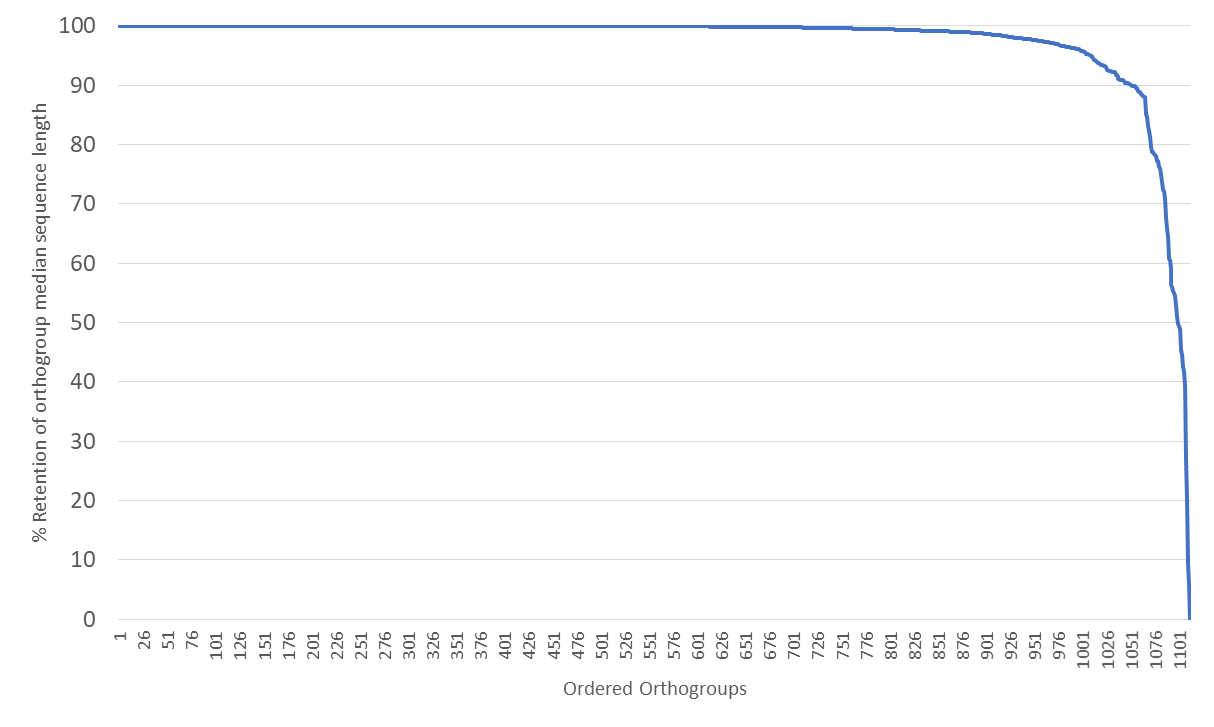


### Supplementary Figure 1. Percent retention of median sequence length in each orthogroup after Gblocks (Talavera and Castresana, 2007) trimming of orthogroup alignments.


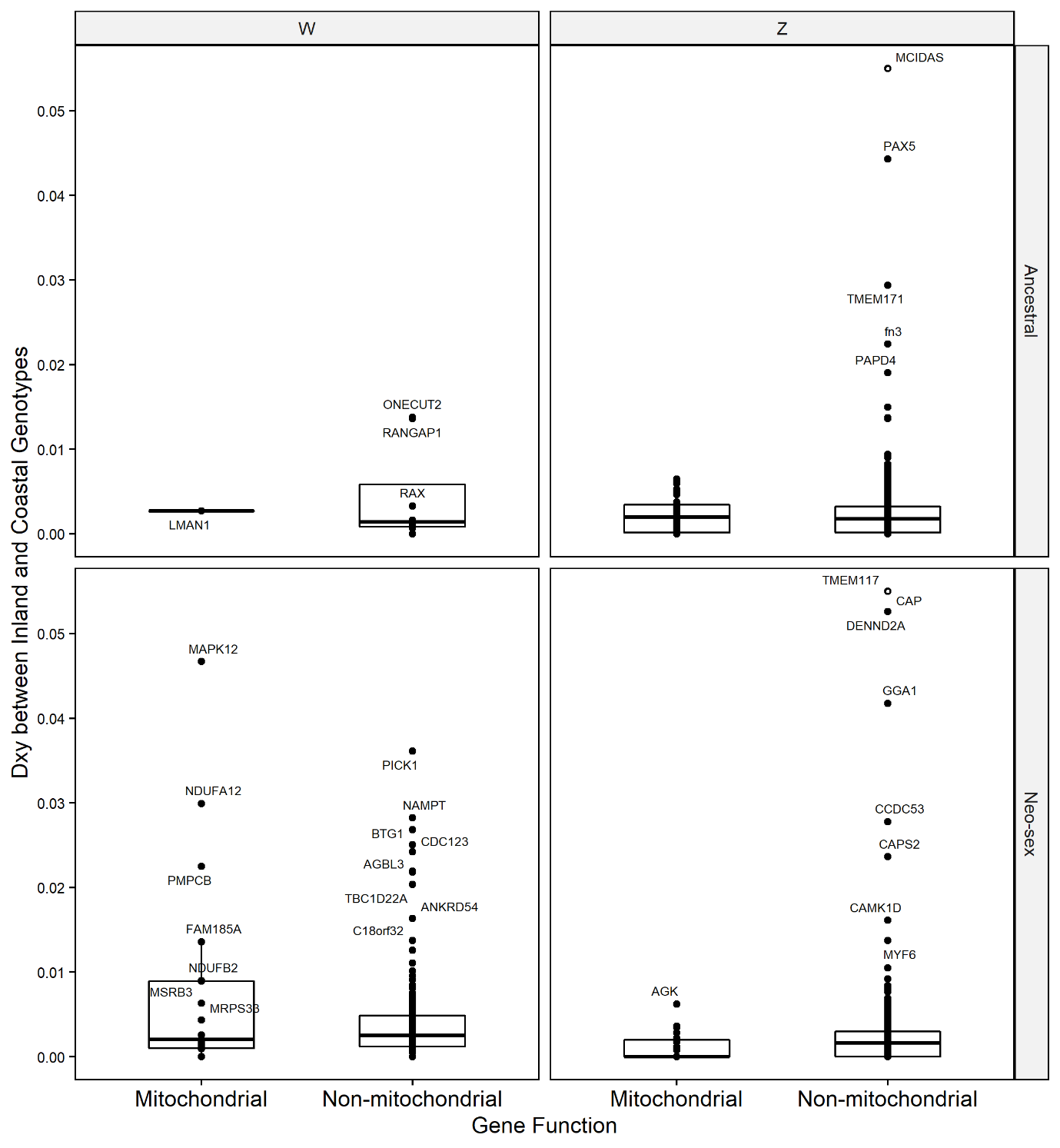


### Supplementary Figure 2. Distributions of sequence divergence (Dxy) values calculated between inland and coastal EYR genes. Genes were partitioned by W versus Z sex linkage (left and right facets), ancestral/neo-sex genomic location (top and bottom facets), and mitochondrial vs non-mitochondrial gene function (left and right within facets). Genes with Dxy values outside the plot scale limits are depicted with open circles. Linear mixed models fit over ancestral and neo-sex genes separately did not detect any significant differences between the distributions of Dxy of N-mt and non-N-mt genes, regardless of sex linkage. Genes exhibiting the highest Dxy values for each category are labelled.


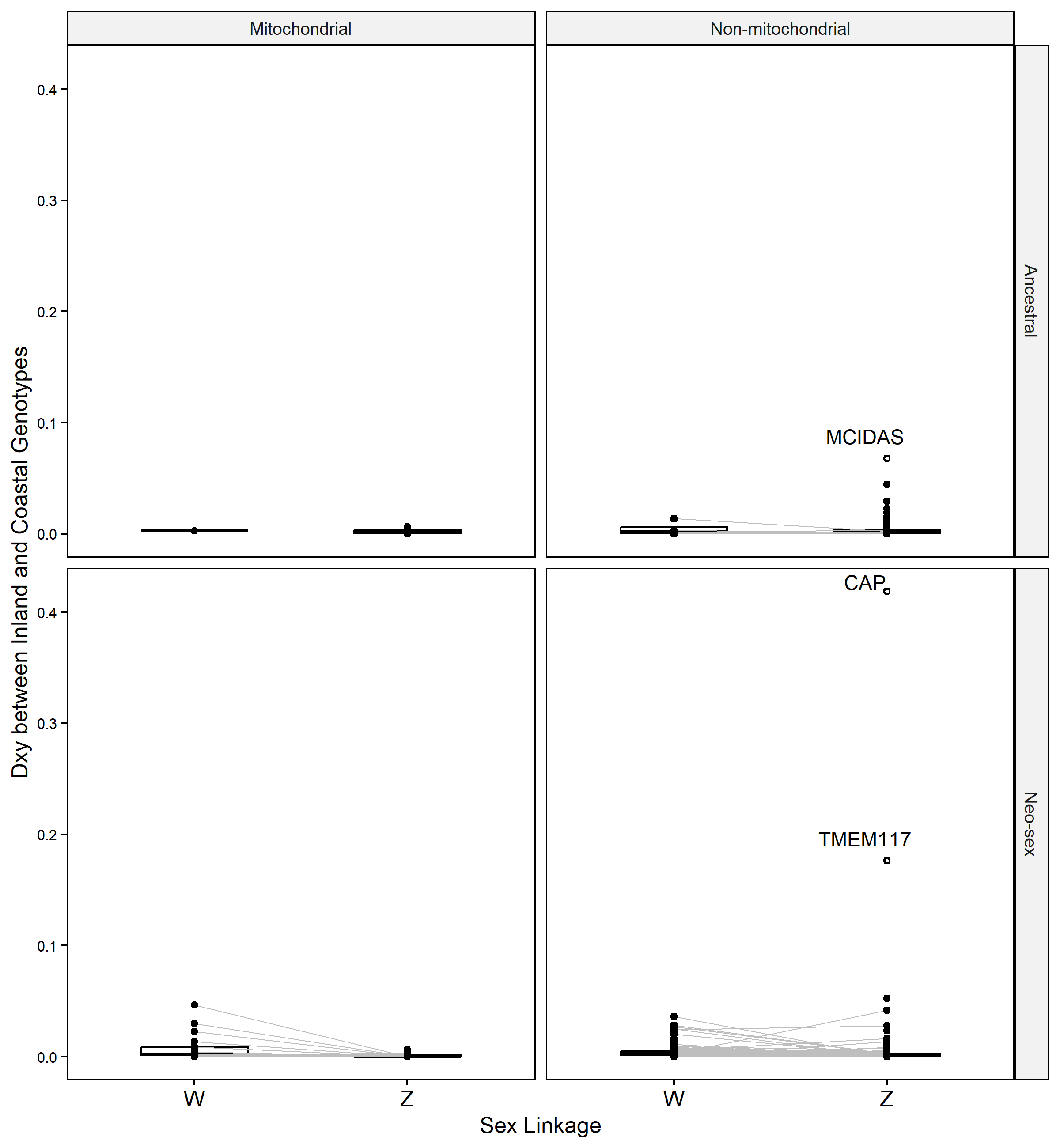


### Supplementary Figure 3. Distributions of sequence divergence (Dxy) values calculated between inland and coastal EYR genes, partitioned by gene function. This version of main Figure 3 is identical apart from an untruncated y-axis to depict extreme outlier Dxy values of some genes.


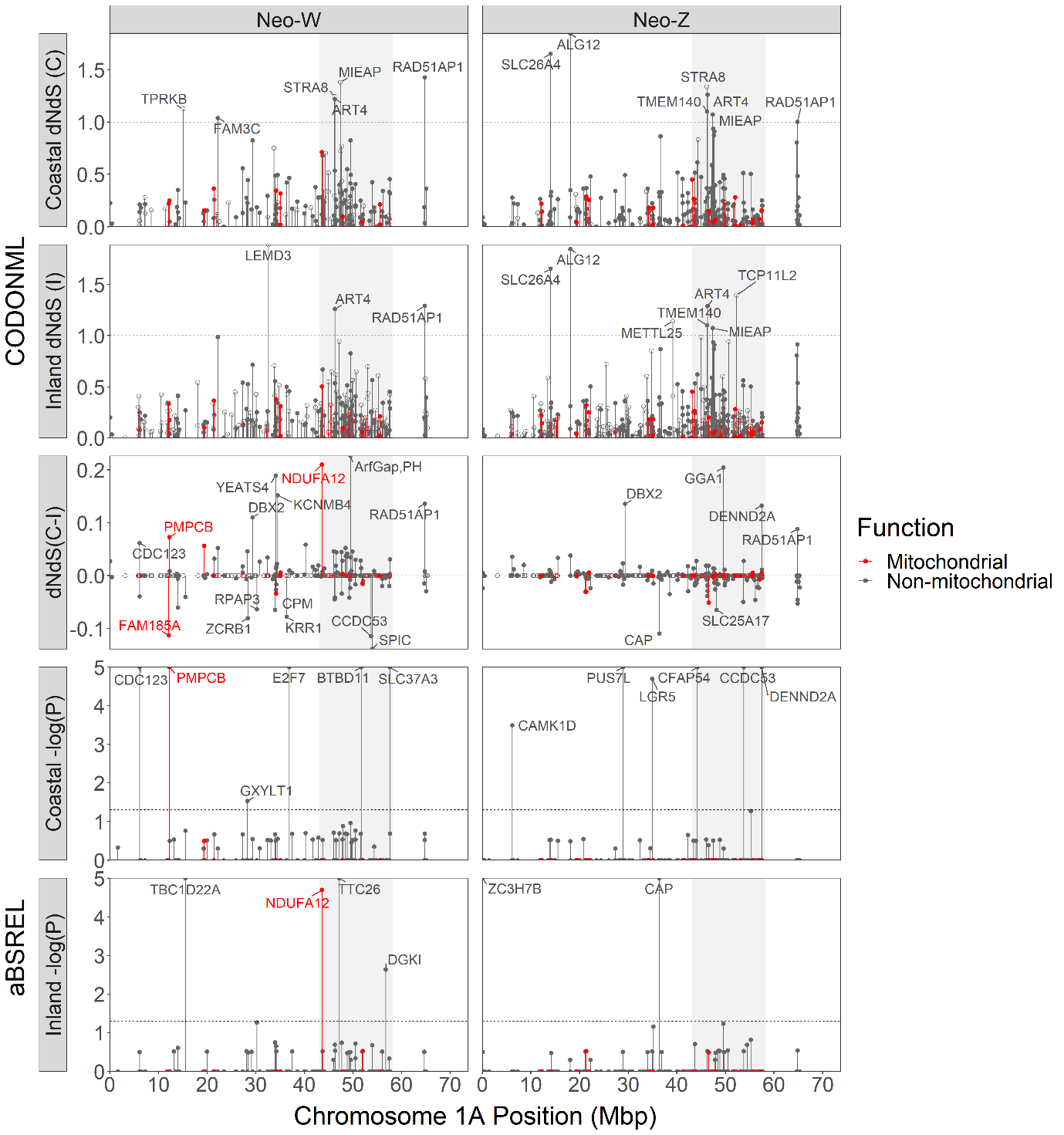


### Supplementary Figure 4. Manhattan plots of N-mt and non-N-mt genes (red and black respectively) located on neo-W and neo-Z chromosomes (left and right columns). Plots depict CODONML gene-wide dNdS estimates (top two rows), differences in CODONML dNdS rate estimates between EYR lineages (middle row, dNdS(C-I)), and –log(P) values for aBSREL tests for positive selection. Zebra Finch chromosome 1A is used to provide reference position genome coordinates for each gene and the region of high between-lineage nuclear differentiation found in Morales et al. (2018) is indicated by the greyed out region. This version of main Figure 4 is identical apart from labelling all putative CODONML and aBSREL hits.

### References

Gurevich A, Saveliev V, Vyahhi N, Tesler G (2013). QUAST: Quality assessment tool for genome assemblies. *Bioinformatics (Oxford, England)* **29**(8)**:** 1072-1075.

Smith AC, Robinson AJ (2018). Mitominer v4.0: An updated database of mitochondrial localization evidence, phenotypes and diseases. *Nucleic Acids Res* **47**(D1)**:** D1225-D1228.

Smith MD, Wertheim JO, Weaver S, Murrell B, Scheffler K, Kosakovsky Pond SL (2015). Less is more: An adaptive branch-site random effects model for efficient detection of episodic diversifying selection. *Molecular Biology and Evolution* **32**(5)**:** 1342-1353.

Talavera G, Castresana J (2007). Improvement of phylogenies after removing divergent and ambiguously aligned blocks from protein sequence alignments. *Systematic Biology* **56**(4)**:** 564-577.

Wertheim JO, Murrell B, Smith MD, Kosakovsky Pond SL, Scheffler K (2015). RELAX: Detecting relaxed selection in a phylogenetic framework. *Molecular Biology and Evolution* **32**(3)**:** 820-832.

Yang Z (2007). PAML 4: Phylogenetic analysis by maximum likelihood. *Molecular Biology and Evolution* **24**(8)**:** 1586-1591.
