# Supplementary material for: Accelerated differentiation of neo-W nuclear-encoded mitochondrial genes between two climate-associated bird lineages signals potential co-evolution with mitogenomes
Source: Heredity (Edinb). 2024 Aug 22;133(5):342–54. doi: 10.1038/s41437-024-00718-w (PMC11527876; doi:10.1038/s41437-024-00718-w)
Supplement: Supplementary file 2 — S2. Supplementary Methods [file 41437_2024_718_MOESM2_ESM.docx]

**Supplementary methods for:**

**Accelerated differentiation of neo-W nuclear-encoded mitochondrial genes between two climate-associated bird lineages signals potential co-evolution with mitogenomes**

Gabriel Weijie Low, Alexandra Pavlova, Han Ming Gan, Meng-Ching Ko, Keren R Sadanandan, Yin Peng Lee, Nevil Amos, Lana Austin, Stephanie Falk, Damian Dowling, Paul Sunnucks

**Additional methods are detailed here, where referred to in the main manuscript.**

*Long-read sequencing for VIC030 genome assembly*

Long-read data for the VIC030 genome assembly was obtained using two approaches. First, an Oxford Nanopore sequencing library was constructed from G-tube fragmented (∼8 kb) genomic DNA, using the LSK108 library preparation kit (Oxford Nanopore, Oxford, UK). Sequencing was performed on 2 MinIONR9.4.1 flowcells for 48 hours, followed by fast5 base-calling using Albacore v2.0.1 to yield ~2x sequencing depth. In addition, a PacBio library was prepared following the manufacturer’s protocol and sequenced on the PacBio RSII SMRT platform at the University of Melbourne to obtain ~10x sequencing depth.

*RNA-seq library preparation and sequencing*

Total RNA was extracted from each sampled tissue of female M19.32.1 and male M19.32.2 using the Quick-RNA MiniPrep Kit (Zymo Research, Irvine, California, USA) according to the manufacturer’s protocols. RNA was quantified using a Qubit 4.0 Fluorometer (Invitrogen, USA) and quality checked with a TapeStation 4200 System (Agilent, Santa Clara, CA, USA). We pooled equimolar amounts of RNA from each tissue type for each individual, and prepared each library from 500 ng of RNA (in a volume of 50 µL) using the NEBNext® Ultra™ II Directional RNA Library Prep Kit for Illumina® (New England Biolabs, USA) according to the manufacturer's protocol, except including a 5-fold dilution of the NEBNext adaptor for Illumina (provided at 15 μM) in dilution buffer (10 mM Tris-HCI, 10 mM NaCl) (Astral Scientific, Australia). We ran 8 cycles of PCR enrichment for adaptor-ligated DNA, before re-quantifying and estimating the fragment size distribution of the prepared libraries with the Qubit 4.0 Fluorometer and TapeStation 4200 System. We enzymatically treated 2 µL of each library with Illumina Free Adapter Blocking Reagent (Illumina, San Diego, CA), and then preliminarily sequenced it on the MiniSeq Sequencer (2 x 150 bp paired-end reads) (Illumina, San Diego, CA) to obtain the read distribution of each sample. Each library was then re-pooled to equimolar concentrations, enzymatically treated, denatured and normalized to 2 nM. Finally, the pooled library was sequenced on the NovaSeq 6000 Sequencer (2 x 150 bp paired end reads) (Illumina, San Diego, CA) at Deakin Genomics Centre, Australia.

*Sex linkage detection in genomic scaffolds*

We adopted a previously-used approach to detect sex-linkage in scaffolds, based on kmer-matching and differential mapping-coverage between sets of male and female WGS reads in each lineage (Gan *et al*, 2019). Specifically, unique kmers (nucleotide strings of length k) are expected to be present on W-linked scaffolds if they are diverged from gametologous Z-linked scaffolds. Scaffolds with kmers detected only in females are presumed W-linked. In addition, we expected that sequencing read-depth of sex-linked chromosomes relative to autosomes differs between sexes: males should exhibit diploid-equivalent depth in Z-inherited regions (including neo-Z, if present) and zero depth in W-inherited regions (including neo-W, if present), whereas females should exhibit haploid-equivalent depth in Z- and in W-inherited regions. Finally, we expect W-linked scaffolds to be poorly covered by sequence reads from males (near zero coverage, or at least significantly lower than that of females even in sections of neo-W sequence that are relatively undiverged from neo-Z).

We first cleaned raw WGS reads by conducting poly-G, adaptor- and quality-trimming with fastp v0.20.0 (Chen *et al*, 2018) for each of the 36 resequenced individuals. Trimmed sequences from males and females of each lineage were processed and aligned against their respective genome assemblies with BWA-MEM v0.7.17 (Li, 2013) and SAMtools v1.9 (Li *et al*, 2009). We verified the sex assignment of sequenced individuals by mapping their resequencing data to known EYR CHD-W and CHD-Z genes (GenBank accessions KC466842.1 & KC466846.1 respectively; (Pavlova *et al*, 2013)) using BLASTN 2.2.31+ (Altschul *et al*, 1990). We also confirmed the mitolineage of each resequenced individual by mapping their reads against the inland mitogenome, extracted from the EYR054 genome assembly (Gan *et al*, 2019), with BWA-MEM and calling the consensus sequence with the SAMtools package.

We standardized each resequenced individual’s read depth by using SAMtools to randomly subset ~66 million reads, the number of reads in the least-sequenced sample. We then pooled male and female reads of inland and coastal EYR lineages separately, and calculated their mean read depth and horizontal sequencing coverage along each scaffold of their respective (inland or coastal) genomes with BamStats04 from the JVarkit package suite (Lindenbaum, 2015). We then calculated the male to female ratios of both metrics (MFavedep and MFcov respectively) for each scaffold.

We calculated kmer frequencies for all 16-bp kmers separately across males and females for each mitolineage with Jellyfish v1.1.12 (Marçais and Kingsford, 2011), following published protocols (Gan *et al*, 2019). We then calculated the percentage of unmatched single-copy kmers (PUK) between pooled EYR male reads from each lineage and their respective female reference genome with the perl script YGS.pl (Carvalho and Clark, 2013), which in the process also matched female reference kmers against pooled female reads to validate male-female kmer comparisons.

We excluded all poorly sequenced/assembled scaffolds with <5 kb length, <5% horizontal sequencing coverage by WGS reads, or <5 mean depth per base, and then summarized the mean male-to-female WGS map depth, horizontal coverage ratios, and PUK (indicating W-linkage). We clustered scaffolds based on these summarized metrics using a Principal Component Analysis (PCA) built with FactoMineR (Lê *et al*, 2008) and visualized the results as an interactive biplot with R packages factoextra (Kassambara and Mundt, 2017), ggplot2 (Wickham, 2016), and plotly (Sievert, 2020). We expected that W- and Z-linked scaffolds would cluster separately from autosomal scaffolds, and ascertained that the EYR CHD-W and CHD-Z genes were present on scaffolds in separate clusters in PC1 vs PC2 space. We built a frequency distribution of scaffold PC1 eigenvalues for both the coastal and inland genome assemblies (0.15 bin width, representing ~88 and ~93% of variation respectively) to visualize the separation of clusters along this main axis.

Additionally, we assessed the power of our chosen sex-linked metrics to assign sex linkage to given genomic scaffolds by performing a linear discriminant analysis with cross-validation, using the ‘MASS’ (Venables and Ripley, 2002) and ‘caret’ (Kuhn 2008) packages in R. We estimated prediction accuracy by using our linear discriminant model to predict the sex linkage of all scaffolds, as well as just the subset of scaffolds that were assigned as sex-linked through the PCA-based analysis.

*Orthogroup (OG) alignment, sex-linked sequence fragment rescue, and site filtering*

We filtered out all OrthoFinder-generated (Emms and Kelly, 2019) OGs that did not contain transcripts annotated on sex-linked scaffolds in coastal as well as inland EYR genomes, and further retained only those containing at least two outgroup representatives. We then performed a length-based sequence filter on the sequences in each OG, keeping only sequences that were >=50% of each OG’s mean base length. To subsequently filter out OGs with high incidences of paralogs, we retained only those that contained at most 4 total sequences for every 3 outgroup taxa present. All OGs were aligned using MACSEv2 (Lindenbaum, 2015) to generate amino acid and nucleotide alignments. We trimmed non-homologous sites with HMMCleaner (Di Franco *et al*, 2019) and retained only sequences with >=50% of their pre-filter input length.

Repetitive regions in sex chromosomes tend to reduce scaffold contiguity during assembly and potentially lead to erroneous fragmentation of genes during annotation (Peona *et al*, 2021). To rescue Z- and W-linked EYR sequences in each OG that were erroneously assembled into separate scaffolds in this manner and thereafter annotated as separate transcripts, we devised criteria by which to merge them. We first calculated the alignment overlap length of all possible pairwise combinations of coastal and inland EYR sequences in each aligned OG separately with a custom script. We then performed a stepwise merger of fragments by collapsing individual fragments into single consensus sequences, beginning with the pair of candidate fragments that produced the longest resultant post-merger length while having an overlap length < 5% of the shorter candidate’s length. Overlap lengths were recalculated after every merger, continuing recursively until no fragment pairs met the criteria, at which point only the longest composite sequence of each sex-linkage and EYR lineage was retained in an OG. We ran a post-merger sequence-length filter of all sequences in each OG to further enrich our datasets for informative sites, keeping only sequences in each OG with internal gap-to-base ratios <1 or within 2.5 median absolute deviations of the mean gap-to-base ratio for that orthogroup.

We used Gblocks (Talavera and Castresana, 2007) to remove all codon sites with >50% missing data across taxa from each orthogroup. We then inspected the distribution of median sequence lengths in each OG after trimming to determine the point at which sequence-length retention drops markedly, and kept orthogroups with ≥ 85% sequence-length retention (Supplementary Figure 1). To reduce the number of paralogs or incorrectly assigned sex-linked genes, we accepted only single representatives of autosomal sequences for each EYR individual in each orthogroup, and removed all orthogroups without any sex-linked EYR sequence representation remaining.

## References

Altschul SF, Gish W, Miller W, Myers EW, Lipman DJ (1990). Basic local alignment search tool. *Journal of Molecular Biology* **215**(3)**:** 403-410.

Carvalho AB, Clark AG (2013). Efficient identification of Y chromosome sequences in the human and drosophila genomes. *Genome Research* **23**(11)**:** 1894-1907.

Chen S, Zhou Y, Chen Y, Gu J (2018). Fastp: An ultra-fast all-in-one fastq preprocessor. *Bioinformatics (Oxford, England)* **34**(17)**:** i884-i890.

Di Franco A, Poujol R, Baurain D, Philippe H (2019). Evaluating the usefulness of alignment filtering methods to reduce the impact of errors on evolutionary inferences. *BMC Evolutionary Biology* **19**(1)**:** 21.

Emms DM, Kelly S (2019). Orthofinder: Phylogenetic orthology inference for comparative genomics. *Genome Biology* **20**(1)**:** 238.

Gan HM, Falk S, Morales HE, Austin CM, Sunnucks P, Pavlova A (2019). Genomic evidence of neo-sex chromosomes in the Eastern Yellow Robin. *GigaScience* **8**(9).

Kassambara A, Mundt F (2017). Package ‘factoextra’: Extract and visualize the results of multivariate data analyses. **76**.

Kuhn M (2008). Building predictive models in R Using the caret package. *Journal of Statistical Software* **28**(5), 1–26. <https://doi.org/10.18637/jss.v028.i05>

Lê S, Josse J, Husson F (2008). Factominer: An R package for multivariate analysis. *2008* **25**(1)**:** 18.

Li H, Handsaker B, Wysoker A, Fennell T, Ruan J, Homer N et al. (2009). The sequence alignment/map format and SAMtools. *Bioinformatics* **25**(16)**:** 2078-2079.

Li H (2013). Aligning sequence reads, clone sequences and assembly contigs with BWA-MEM. *arXiv preprint arXiv:13033997*.

Lindenbaum P (2015). Jvarkit: Java-based utilities for bioinformatics.

Marçais G, Kingsford C (2011). A fast, lock-free approach for efficient parallel counting of occurrences of k-mers. *Bioinformatics* **27**(6)**:** 764-770.

Pavlova A, Amos JN, Joseph L, Loynes K, Austin JJ, Keogh JS et al. (2013). Perched at the mito-nuclear crossroads: Divergent mitochondrial lineages correlate with environment in the face of ongoing nuclear gene flow in an Australian bird. *Evolution* **67**(12)**:** 3412-3428.

Peona V, Blom MPK, Xu L, Burri R, Sullivan S, Bunikis I et al. (2021). Identifying the causes and consequences of assembly gaps using a multiplatform genome assembly of a bird-of-paradise. *Molecular Ecology Resources* **21**(1)**:** 263-286.

Sievert C (2020). *Interactive web-based data visualization with R, plotly, and Shiny*. Chapman and Hall/CRC Press.

Talavera G, Castresana J (2007). Improvement of phylogenies after removing divergent and ambiguously aligned blocks from protein sequence alignments. *Systematic Biology* **56**(4)**:** 564-577.

Venables WN, Ripley BD (2002) *Modern Applied Statistics with S. Fourth Edition*. Springer, New York. ISBN 0-387-95457-0

Wickham H (2016). *ggplot2: Elegant graphics for data analysis*. Springer.
